# Overcoming multidrug-resistant lung cancer by mitochondrial-associated ATP inhibition using nanodrugs

**DOI:** 10.1186/s12951-023-01768-8

**Published:** 2023-01-12

**Authors:** Jun-Young Park, Gyu-Ho Lee, Kwai Han Yoo, Dongwoo Khang

**Affiliations:** 1 Lee Gil Ya Cancer and Diabetes Institute, Gachon University, Incheon, 21999 South Korea; 2 Department of Health Sciences and Technology, GAIHST, Gachon University, Incheon, 21999 South Korea; 3 Department of Physiology, College of Medicine, Gachon University, Incheon, 21999 South Korea; 4 Department of Internal Medicine, Gachon University Gil Medical Center, College of Medicine, Incheon, 21565 South Korea

**Keywords:** Carbon nanotube, Doxorubicin, Endosomal escape, Multidrug resistant cell, Small cell lung cancer, Mitochondrial damage

## Abstract

Despite the development of therapeutic modalities to treat cancer, multidrug resistance (MDR) and incomplete destruction of deeply embedded lung tumors remain long-standing problems responsible for tumor recurrence and low survival rates. Therefore, developing therapeutic approaches to treat MDR tumors is necessary. In this study, nanodrugs with enhanced intracellular drug internalization were identified by the covalent bonding of carbon nanotubes of a specific nano size and doxorubicin (DOX). In addition, carbon nanotube conjugated DOX (CNT-DOX) sustained in the intracellular environment in multidrug-resistant tumor cells for a long time causes mitochondrial damage, suppresses ATP production, and results in the effective therapeutic effect of drug-resistant tumors. This study identified that H69AR lung cancer cells, an adriamycin (DOX) drug-resistant tumor cell line, did not activate drug resistance function on designed nano-anticancer drugs with a specific nano size. In summary, this study identified that the specific size of the nanodrug in combination with DOX overcame multidrug-resistant tumors by inducing selective accumulation in tumor cells and inhibiting ATP by mitochondrial damage.

**Supplementary Information:**

The online version contains supplementary material available at 10.1186/s12951-023-01768-8.

## Introduction

Small cell lung cancer (SCLC) is an intractable cancer. It ranks sixth in cancer-related mortality and accounts for approximately 15% of all lung cancers [1]. Unfortunately, the 5-year survival rate of SCLC is less than 7% [2, 3]. In clinical practice, chemotherapy is the mainstream treatment, and although most patients have a high response rate at the initial stage of treatment, it causes recurrence, and the therapeutic effect is poor because of the development of drug resistance due to repeated treatment [4, 5]. The lack of effective follow-up therapy after relapse has resulted in poor outcomes in patients who have failed treatment. Drug resistance is another major obstacle that affects the effectiveness of chemotherapy and leads to poor clinical outcomes. Therefore, new strategies for the treatment of drug-resistant SCLC are urgently necessary in the clinical setting [6].

Numerous mechanisms, such as mutations of drug targets and the production of cancer stem cells (CSCs), are involved drug resistance [7]. Nonetheless, ATP-binding cassette (ABC) efflux transporters, including multidrug resistance protein 1 (mrp-1), P-glycoprotein (P-gp), and breast cancer resistance protein, were found to mediate drug efflux and to be closely related to multidrug resistance (MDR) [8]. P-glycoprotein (P-gp), one of the ATP-dependent efflux pumps and overexpressed in multidrug-resistant (MDR) tumors, is an important protein in the cell membrane that removes many foreign substances from cells and is widely distributed in various tissues such as the liver, pancreas, kidney, and colon [9]. As ATP binds to the protein's cytoplasm, ATP is hydrolyzed at each binding to release ADP, and ATP re-binds to activate the pump [10]. Regarding the expression and function of P-gp in cancer cells, P-gp has been intensively studied at the transcriptional level, and various transcription factors such as p53 and NF-κB are directly regulated by binding to the promoter region of P-gp [11]. In addition, multidrug resistance protein-1 (mrp-1), a type of ABC protein that is an ATP-dependent membrane protein, is one of the causes of multidrug-resistant cancer cells; this protein also acts as it is expressed in the cell membrane [12, 13]. The mrp-1 has been found to mediate adriamycin (i.e., DOX), irinotecan, methotrexate, and floxuridine resistance as more drug is pumped from cancer cells due to overexpression of mrp-1 [14, 15]. Therefore, it represents another important strategy in the pursuit of methods that inhibit the drug efflux pump, in addition to combination therapies such as photo/chemotherapy, which have shown excellent antitumor effects, especially for drug-resistant cancers [16–18]. In the literature, methods that directly inhibit mrp-1 using specific pump inhibitors or directly target cancer through drug delivery systems such as nanomaterials have been used to overcome multidrug-resistant cancer cells [19, 20].

Types of nanocarriers used for drug delivery include carbon nanotubes (CNTs), liposomes, gold nanoparticles, polymer micelles, dendrimers, and magnetic nanoparticles [21]. CNTs are circular in graphene structure and have a structure with three carbon atoms bonded to the carbon atom. These CNTs have high durability and stability, and are used as carriers by binding to many drugs [22]. Particularly notable is that CNTs have been mainly used in nanocarrier drug delivery systems due to the advantage of being easily functionalized by surface modification through non-covalent and covalent bonds [23]. The surface of CNTs can be oxidized for surface functionalization and coated with amphiphilic polymers or surfactants for efficient cellular uptake through specific endocytotic pathways [24]. Due to its stability by strength and excellent hardness, CNTs are widely used as drug carriers for biomedicine, genetic engineering, artificial implantation, imaging, cancer treatment, antioxidant activity, and biosensing [25]. On the other hand, cylindrical nanostructures, including CNTs, were considered to have a detrimental effect as a source of lung aerosols [26]. For example, the “needle-like” shape of CNTs, coupled with a high aspect ratio (length-to-width ratio), hydrophobicity, and bio-persistence, has raised concerns about lung toxicity [27]. However, this type of nanodrug can be a highly effective anti-lung cancer agent that specifically targets lung tumors because it selectively accumulates in lung tumor tissues [28].

**Fig. 1:**
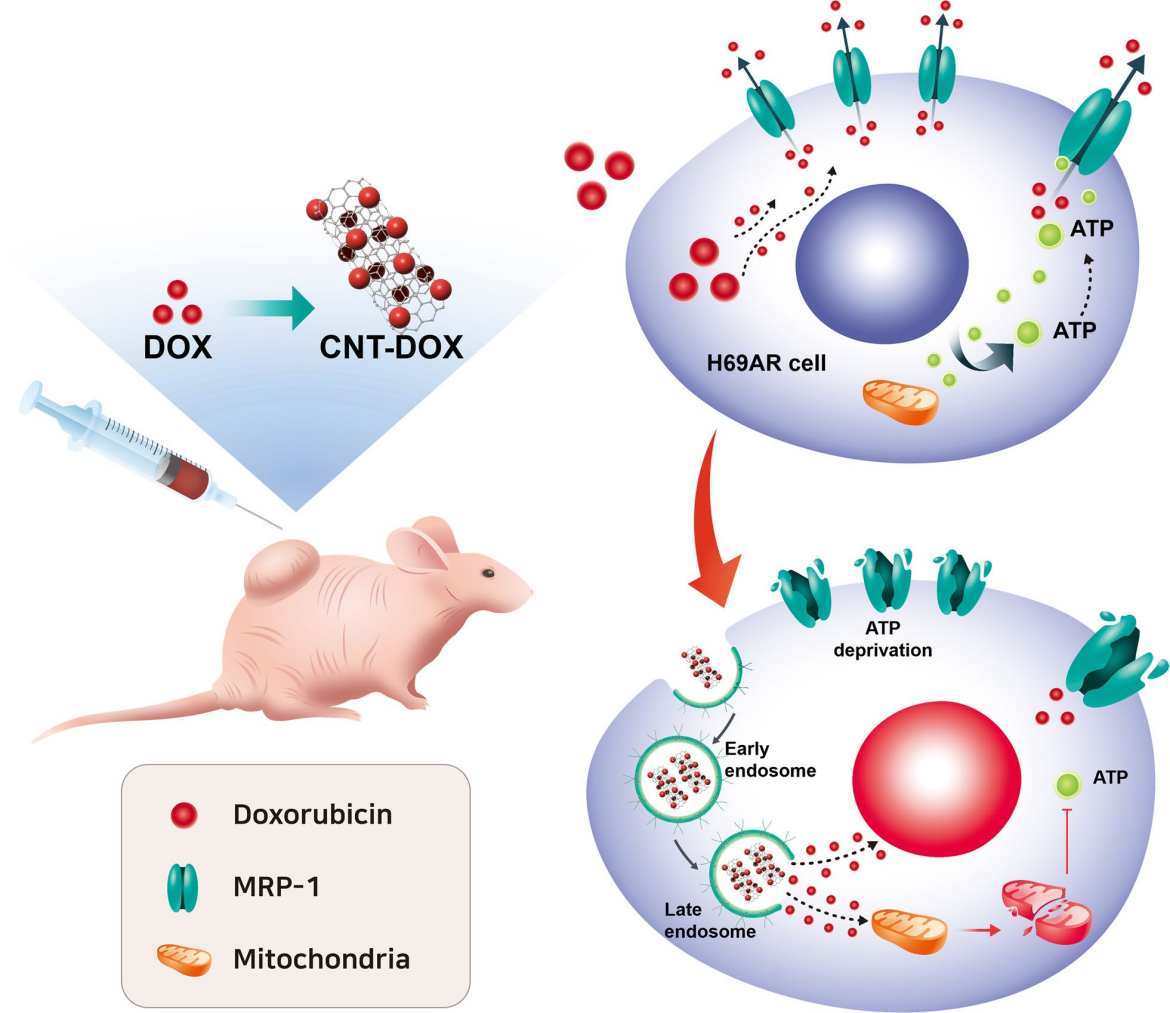
Schematic of therapeutic effect in multidrug-resistant tumor. The schematic represents mitochondrial membrane disruption of multidrug-resistant lung cancer cells (H69AR cells) by using carbon nanotubes conjugated with doxorubicin, which provides ATP deprivation and resulted in nullifying efflux function of multidrug-resistant tumor and finally, induced cancer apoptosis

The purpose of this study was to overcome the efflux of resistant cancer cells by using CNT-based nanodrugs with a specific size. In multidrug-resistant small cell lung cancer, 60–100 nm of CNT-DOX was uptaked and transported through endocytosis and did not encounter efflux compared with DOX. In addition, the uptake of the nanodrug accumulated in a prolonged manner inside the DOX resistance cancer cells. Specifically, CNT-DOX induces mitochondrial damage and inhibits ATP production. Consequently, this study identified the apoptotic effect of multidrug-resistant small-cell lung cancer via mitochondrial damage in resistant cancer cells and nullifying the function of mrp-1 (Fig. 1).

## Materials and methods

### CNT fabrication

Sizes of CNT were purchased from SES Research. CNTs were heated at 300 ℃ for 2 h to evaporate the water vapor. Next, 400 mg CNT were added to 180 ml sulfuric acid and 60 ml nitric acid and reacted for 99 min under sonication. After 99 min, the reaction was conducted at 55 degrees/200 RPM for 48 h. After 48 h, 20 ml 240 ml CNT was mixed with 2 L of DW and diluted. Subsequently, 300 ml of diluted CNT was added and filtered using a 0.1 ~ 0.2 μm PTFE membrane filter purchased from Merck Millipore. DW (25 ml) was added to the filtered CNTs and washed once. After removing the filtered CNT filter paper, it was placed on a 150 dish, stored for 2 d at 60 ℃/0.07 vacuum, dried, collected, transferred to a 2 ml piece of e-tube, and stored at -20 ℃ until use.

### Covalently conjugated CNT with DOX

CNT (40 mg) was placed in 10 ml DW and sonicated for 10 min. Centrifugation was performed at 4000 RPM for 10 min, and DW washing was performed 3 times in total. After washing, 40 mg CNT and 20 ml pH 6 MES buffer were warmed to 50 ℃, and 920 mg N-hydroxysuccinimide (NHS) and 10 mL pH 6 MES buffer were added; next, CNT and NHS were mixed, followed by sonication for 5 min. First, 900 mg 1-Ethyl-3-(3-dimethylaminopropyl) carbodiimide (EDC) was reacted by rotation at room temperature for 1 h. After the reaction was completed, centrifugation was performed at 4000 RPM for 15 min, and washing was performed three times using 10 ml MES (pH 6). For DOX, when washing CNTs with 10 ml pH 6 MES, 40 mg CNTs was pre-dissolved in a weight ratio of 1:1 in 5 ml pH 6 MES, stored at 4 ℃ after washing, released in 5 ml pH 6 MES, and mixed with DOX at 4 ℃ overnight. After the overnight reaction, washing was performed three times using 10 ml MES buffer (pH 6 MES buffer was warmed to 50 ℃ to remove DOX that did not adhere to CNTs; next, CNT DOX was released with 2 ml DW and collected in a 2 ml e-tube. CNT-DOX was diluted 50-fold in DW in a UV–vis cuvette, and the total volume was 2 ml. In the measured CNT DOX, DOX was measured at 480 nm and CNT at 700 nm. DOX loading (%) = weight of DOX attached to the CNT / Weight of CNT × 100.

### Cell cultures

H69AR (non-SCLC, CRL-11351™) cells supplemented with 10% FBS (16000-044, Gibco) and 1% penicillin streptomycin (15140-163, Gibco) were maintained in RPMI 1640 medium (11875-093, Gibco) and were cultured in a humidified incubator with 5% CO_2_ at 37 ℃.

### Uptake analysis of DOX resistance cancer cells

Poly D lysine coating was conducted by placing a cover glass in a 150 dish, putting 70% ethanol, and irradiating via UV for 1 d. After 1 d, using DW, washing was performed four times for 5 min each. After washing, the cover glass was transferred to a 24-well plate. The transferred cover glass was irradiated with UV until the phosphate-buffered saline (PBS) disappeared in 24 wells; when PBS disappeared, it was stored at 4 ℃ MV. Poly D lysine-coated cover glass was placed in a 24-well plate, and RPMI-1640 + 10% FBS + 1% P/S was added and preincubated for 30 min. After seeding 1 × 10^5^ cells, the next-day inhibitors (macropinocytosis: EIPA, clathrin: CPZ, caveolin: GEN) were treated with 20 μM, 25 μM, and 200 μM at each concentration for 1 h before drug treatment. After treating CNT DOX 500 ng/ml, incubation was conducted for each hour; after removing the media, it was washed three times with PBS, 500 μl of 4% paraformaldehyde (PFA) was added, and this fixed for 1 d. After fixation, 5 µL mounting solution was placed on a glass slide, and the cover glass with cells was turned upside down for mounting. After incubation at room temperature for 30 min, the RFP fluorescence intensity was checked using EVOS.

### Viability and apoptosis analysis

Next, 3 × 10^4^ cells of H69AR were dispensed into a 96-well plate (100 ul each). The next day, GEN, a caveolin inhibitor, was treated with 200 μM 1 h before the CNT DOX treatment. CNT DOX was treated with the concentrations of 0.125 μg/ml, 0.25 μg/ml, 0.5 μg/ml, and 1 μg/ml and incubated at 36 ℃ / 5% CO_2_ for 48 h. After 48 h, 100 of 2 mg/ml MTT solution was put into each well and incubated at 36 ℃ and 5% CO_2_ for 2 h. After 2 h of reaction, the solution was removed, and 100 μl dimethyl sulfoxide (DMSO) was added to each well to dissolve the formazan crystals, which were then measured at 570 nm.

For the investigation of the effect of covalent CNT DOX on the apoptosis of H69AR, the cells were aliquoted in 2 ml 8 × 10^5^ cells in a 6-well plate. The next day, GEN, a caveolin inhibitor, was treated with 200 μM 1 h before CNT DOX treatment. After 1 h, 0.5 μg/ml CNT DOX was treated and incubated at 36 ℃ / 5% CO_2_ for 24 h. The medium was transferred to a 15 ml conical tube, and after washing once, 200 μl 1X TE was added, and the cells were removed by reacting for 5 min. The removed cells were placed in a 15 ml conical tube with medium and centrifuged at 1500 RPM for 5 min; next, the supernatant was removed, and the cells were washed once with PBS. Subsequently, 105 μl fluorescence-activated cell sorting (FACS) buffer (1X Annexin V binding buffer 100 μl + Annexin V 5 μl) was added, the pellet was released, and the reaction was conducted at room temperature for 20 min. After 20 min, 400 μl annexin V binding buffer was added to stop the reaction. After centrifugation at 1500 RPM for 5 min, the supernatant was removed, 500 μl was added to release the cells, the cells were transferred to a FACS tube, and the results were confirmed.

### MRP-1 expression

H69AR cells were seeded in 2 ml of 8 × 10^5^ cells in a 6-well plate. The next day, GEN, a caveolin inhibitor, was treated with 200 μM for 1 h before the CNT DOX treatment. CNT DOX was treated with 1 μg/ml and incubated at 36 ℃ / 5% CO_2_ for 2–24 h. After removing the media after 2–24 h, 1X TE treatment was performed to remove the cells, which were then centrifuged at 1500 RPM for 5 min. After removing the supernatant, 100 μl protein lysis buffer (RIPA buffer 100 μl + 1% protein inhibitor 10 μl) was added and transferred to 1.5 ml e-tube. The mixture was then placed on ice and incubated for 30 min. After 30 min, the mixture was centrifuged at 12,000 RPM at 4 ℃ for 30 min, and the supernatant was transferred to a new 1.5 ml e-tube. The extracted protein was quantified using a Bradford (Coomassie) protein assay kit. After protein quantification, 20 μl of the sample was prepared by adding protein at an appropriate concentration, 4X sample buffer and DW, and boiling at 100 ℃ for 5 min. For SDS PAGE gel, 8% was used, and each prepared sample was placed on the gel wall and loaded at 85 V. A semi-dry transfer was used to transfer the loaded protein to the membrane at 25 V for 35 min. Using 5% skim milk in TBS-T, blocking was performed at room temperature for 1 h, and mrp-1 and beta actin antibodies were conjugated at 4 ℃ / overnight. The next day, after removing the 1^st^ antibody, the membrane was washed with TBS-T, incubated with the 2^nd^ antibody (rabbit, mouse) at room temperature for 1 h, washed again with TBS-T, and detected using ECL. Immunodetection was performed using an enhanced chemiluminescence detection kit (34080, Thermo Fisher Scientific) and protein bands were photographed with a LAS4000 Chemi-Doc imager (Fuji-film, Japan). The full-length stain is represented in Figure S5.

### ATP analysis

Total ATP inside the cells was measured using an ATP assay kit (Invitrogen, A22066). Viewing the inside of the cell is performed as follows: the cell is lysed with RIPA buffer to obtain the protein, and subsequently, the protein is quantified using the BCA assay. In a state containing 1 µg of protein in a volume of 10 μl, it was placed into a 96-well plate and mixed with 90 μl reaction buffer in the absence of light, and the result was checked using a VICTOR Nivo Multimode Microplate Reader (PerkinElmer). At this time, the ATP concentration used in the standard curve was 0.0625–2 μM; next, 10 μl was added in the same manner as the protein, and 90 μl reaction buffer was added to construct a calibration curve, to calculate the value of ATP measured in cells.

### Mitochondrial membrane potential changes

The poly D lysine-coated cover glass was treated in RPMI-1640 medium before cells were dispensed; next, 1 × 10^5^ cells of H69AR cells were dispensed (500 μl each). The next day, CNT-DOX was treated with 1 μg/ml and incubated at 37 °C for 24 h. After 20 h, 4 mM H_2_O_2_ as a positive control was treated for 4 h, and the cells were washed twice with PBS. Subsequently, 1 μM JC-1 was dissolved in PBS, dispense 500 μl to each well, and incubated at 37 °C for 30 min. It was removed after 30 min, washed twice with PBS, and 500 μl 4% PFA was added; next, it was fix for 1 d. For the fixed samples, 5 μl mounting solution was dispensed on a glass slide, and the cover glass with cells was turned over for mounting. Mitochondrial damage was confirmed by measuring the fluorescence intensities of RFP and GFP by using an EVOS7000.

### Intracellular trafficking

The poly-D lysine-coated cover glass was transferred to a 24-well plate, and RPMI-1640 + 10% FBS + 1% P/S was added; it was preincubated for 30 min, and subsequently, 5 × 10^4^ cells were aliquoted. The next day, 0.5 µg/ml CNT-DOX (60–100 nm) and DOX were treated for 6, 12, and 24 h. For each time-treated group, the medium was removed, washed with PBS, and 4% PFA was added, and the medium was fixed at 4 °C for 1 d. The fixed sample was washed three times for 10 min with PBS, and 500 μl of 0.5 Triton x-100 was added and treated for 10 min. Next, we added 500 µl 100 mM glycine, react at 4 °C for 10 min, add 500 µl of blocking buffer, and incubate at room temperature for 1 h. After blocking, the 1st antibody EEA-1 and mannose 6-phosphate receptor (M6PR) were incubated at 4 ℃ for 1 d. After washing 3 times with PBS for 10 min, the 2^nd^ antibody (Alexa 488 goat anti-rabbit, Alexa 488 goat anti-mouse) was incubated at room temperature for 1 h. After washing it with PBS three times, mounting was performed to confirm the fluorescence wavelength in EVOS7000.

### Intracellular pH change

The Poly-D lysine-coated cover glass was seeded 5 × 10^4^. The next day, the drug was administered according to time, and the medium was removed the next day and washed with live cell imaging solution (LCIS). The pH rodo AM solution was then added, and this was incubated at 37 °C for 30 min. After 30 min, they were washed with LCIS and fixed with 4% PFA. In the case of a calibration curve, it was diluted with 10 mM nigericin 5 μl and 10 mM valinomycin 5 μl in 10 ml of pH calibration buffer (pH 4.5, 5.5, 6.5, 7.5) before fixing, placed on each wall, incubated at 37 °C for 5 min, washed with LCIS, and washed with 4% PFA fix it.

### H69AR_flu-GFP_ production

H69AR cells were seeded 1 × 10^5^ in a 6-well plate. The next day, 1 ml lentiviral supernatant containing luciferase and GFP, and 2 ml RPMI-1640 without penicillin were exchanged. After 24 h, the medium containing the lentiviral vector was removed, replaced with RPMI-1640 containing penicillin, and cultured for 24 h. The next day, 2 ml RPMI-1640 was added to the appropriate concentration of puromycin, and the cells were cultured. Next, the puromycin of and the appropriate concentration were continuously added for 1–3 weeks and cultured, and it was observed.

### H69AR_flu-GFP_ Xenograft model

Female BALB/c nude-Foxn-1-nu mice were purchased from Orient Bio (Seoul, Korea). After shipment, five mice were housed per cage in a laminar air flow room maintained at a temperature of 22 ± 2 °C with a relative humidity of 55 ± 5%. In addition, all mice were cared in specific-pathogen-free environment with a 12-h light/dark cycle (lights on at 7:00 AM) for 7–14 days, to acclimatize before the experiment. All animal experiments were carried out in accordance with the Guide for the Care and Use of Laboratory Animals of Gachon University (LCDI-2019-0092). H69AR_Fluc-GFP_, a SCLC cell line, was dissolved in 1 × 10^7^ cells in 100 μl PBS and injected between the dorsal endothelium and integument of mice. We confirmed whether the H69AR_Fluc-GFP_ tumor was established in the mice: 150 mg/kg D-luciferin was injected, and the size of the tumor was confirmed by fluorescence intensity measurement and imaging for 1 min by using IVIS.

### In vivo antitumor efficacy

The tumors of the mice were larger than 100 mm^3^ and were divided into five groups (n = 5)—saline, CNT, free DOX, CNT DOX, and CNT DOX + inhibitor (in 100 μl of PBS). They were injected intratumorally (5 mg/kg) once per week. The size of the tumor was checked once per week by IVIS fluorescence intensity.

### Bioluminescence imaging using GFP animal model

Bioluminescence imaging (BLI) was confirmed by measuring the luciferase activity with an in vivo imaging system (IVIS). BLI was checked once per week. Before imaging, 100 μl luciferin (150 mg/kg) was injected into the mouse, and anesthetized with isoflurane (approximately 3% in air). Anesthetized mice were placed in a chamber protected from the light of the IVIS, and measurements were collected for 1 min. Images were captured using Living Image software. BLI averaged the fluorescence intensity on the animal surface.

### H69AR_flu-GFP_ production

H69AR cells were seeded 1 × 10^5^ in a 6-well plate. The next day, 1 ml lentiviral supernatant containing luciferase and GFP and 2 ml RPMI-1640 without penicillin were exchanged. After 24 h, the medium containing the lentiviral vector was removed, replaced with RPMI-1640 containing penicillin, and cultured for 24 h. The next day, 2 ml RPMI-1640 was added to the appropriate concentration of puromycin, and the cells were cultured. Next, the puromycin of and the appropriate concentration were continuously added for 1–3 weeks and cultured, and this was observed.

### Immuno-histological analysis

The H69AR_Fluc-GFP_ xenograft model was sacrificed. After fixing the cancer tissue with 10% neutral buffered formalin, the cancer tissue was dehydrated and embedded in paraffin. Embedding tissue was sectioned at a size of 5 μm, rehydration was conducted, and the hematoxylin and eosin (H&E) and tunnel assays were performed.

### Toxicity analysis

For the analysis of peripheral circulating blood cells, blood was placed in a vial containing heparin (5 unit/ml) and transferred to ice for analysis, and blood cells were automatically counted. In the case of serum, the blood was allowed to coagulate at room temperature without being disturbed and was centrifuged at 2000 × *g* at 4 ℃ for 15 min to remove the clot by transferring the supernatant to a new tube. Alanine aminotransferase (ALT), aspartate aminotransferase (AST), creatinine, and blood urea nitrogen (BUN) were measured using clinical chemistry reagent kits.

## Results and discussion

### Characterization of nanodrug

**Fig. 2:**
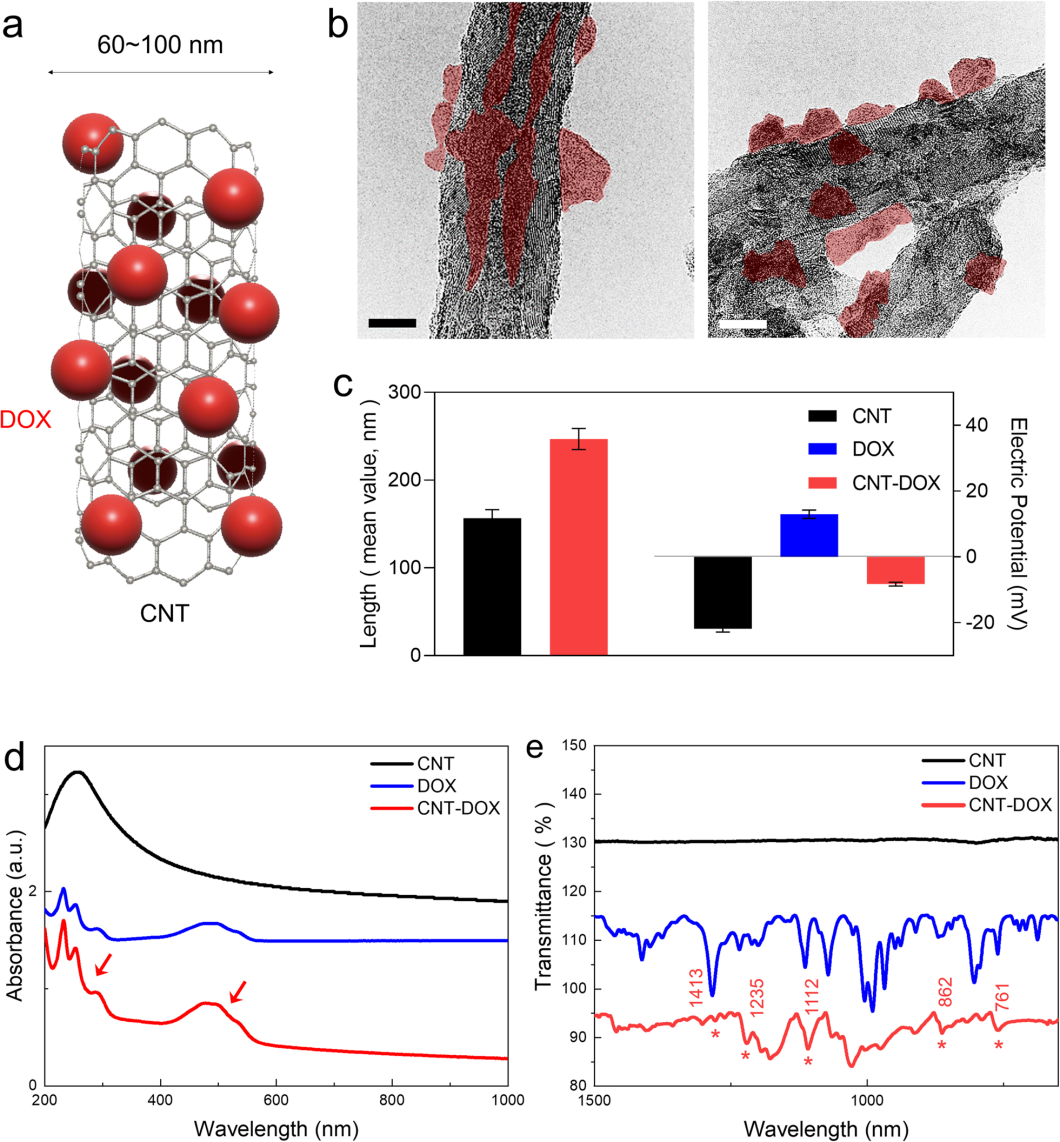
Physiochemical properties. **a** Schematic image of a carbon nanotube (CNT) covalently conjugated with doxorubicin (DOX). **b** Transmission electron microscopic (TEM) images of the CNT conjugated with DOX (CNT-DOX). The scale bar is 20 nm. **c** Mean hydrodynamic sizes (147 ~ 247 nm) and electrical potential of CNT-DOX shows negative potential in aqueous condition. An increase in the average size of a DOX conjugation on CNT and possesses negative charge. **d** UV–vis results show the DOX peak at 480 nm, as identified in CNT-DOX. **e** FT-IR peaks of CNT, DOX, and CNT-DOX. Several identical peaks were observed and analyzed for DOX and CNT-DOX

The physiochemical properties of designed CNT-DOX have been intensively investigated by previous studies [29–31]. Figure 2a represents the transmission electron microscopy (TEM images) of the covalently conjugated CNT with DOX (Fig. 2a) and shows the specific size of CNTs conjugated with DOX through the NHS-EDC reaction (Fig. 2a). The morphology and size of each covalent CNT-DOX with a diameter of 60–100 nm were analyzed using TEM and particle size analysis. TEM images showed that DOX was covalently attached to the CNT (Fig. 2b). A far-field TEM image was analyzed in the supplementary information (Additional file 1: Fig. S1a). In addition, TEM image of pure-CNT with hydrophobic and oxidized CNT with hydrophilic (CNT-COOH) surface structures were analyzed in the supplementary information (Additional file 1: Fig. S2). The lengths of the CNTs were 150 nm and 250 nm after conjugation with DOX. The electric potential analysis confirmed that CNT-DOX had a negative charge in neutral buffer PBS at pH 7.2 (Fig. 2c). Most of the covalently bound nanodrugs showed an average charge between DOX (red) and CNTs that was covalently conjugated with DOX (Fig. 1c). The amount of DOX loaded onto the covalently bound CNT was calculated by analyzing the absorption spectrum. The difference in absorbance peak between the loaded drug (DOX) and CNT at a specific wavelength (480 nm) corresponds to the amount of drug [30]. The weight ratio of loaded DOX on CNT was estimated to be approximately 43% after covalent conjugation (Fig. 2d). Analytical calculation was achieved by determining the difference between DOX-loaded CNT by using UV–vis [32]. In addition, Fourier transform infrared spectroscopy (FTIR) showed the coincidence of peaks for both DOX and covalently conjugated CNT-DOX (Fig. 2e). In addition, strong covalent bonding between DOX and CNT was confirmed by the luminescence quenching ratio using photoluminescence spectroscopy (Additional file 1: Fig. S3). Quenching of covalently conjugated DOX on oxidized CNTs reduced DOX fluorescence by 94.9%, which is comparable to previously reported data for static quenching of DOX via π stacking (80–90% DOX fluorescence reduction) [29] (Additional file 1: Fig. S3). This can be attributed to photoinduced electron transfer (PET) by the strong covalent bond between DOX and CNT, indicating that DOX has undergone a structural change through the coupled electron energy band between oxidized CNT (Additional file 1: Fig. S3). The polydispersity indexes (PDI) of CNT-DOX were less than 0.3 and sustained greater solubility after 7 days after synthesis (Additional file 1: Fig. S1b). Collectively, the TEM, particle size and electric potential measurement, UV–vis, and FTIR analysis clearly showed that DOX was successfully covalently conjugated to the CNTs (Fig. 2b-e).

### Intracellular uptake and clearance of nanodrug

**Fig. 3:**
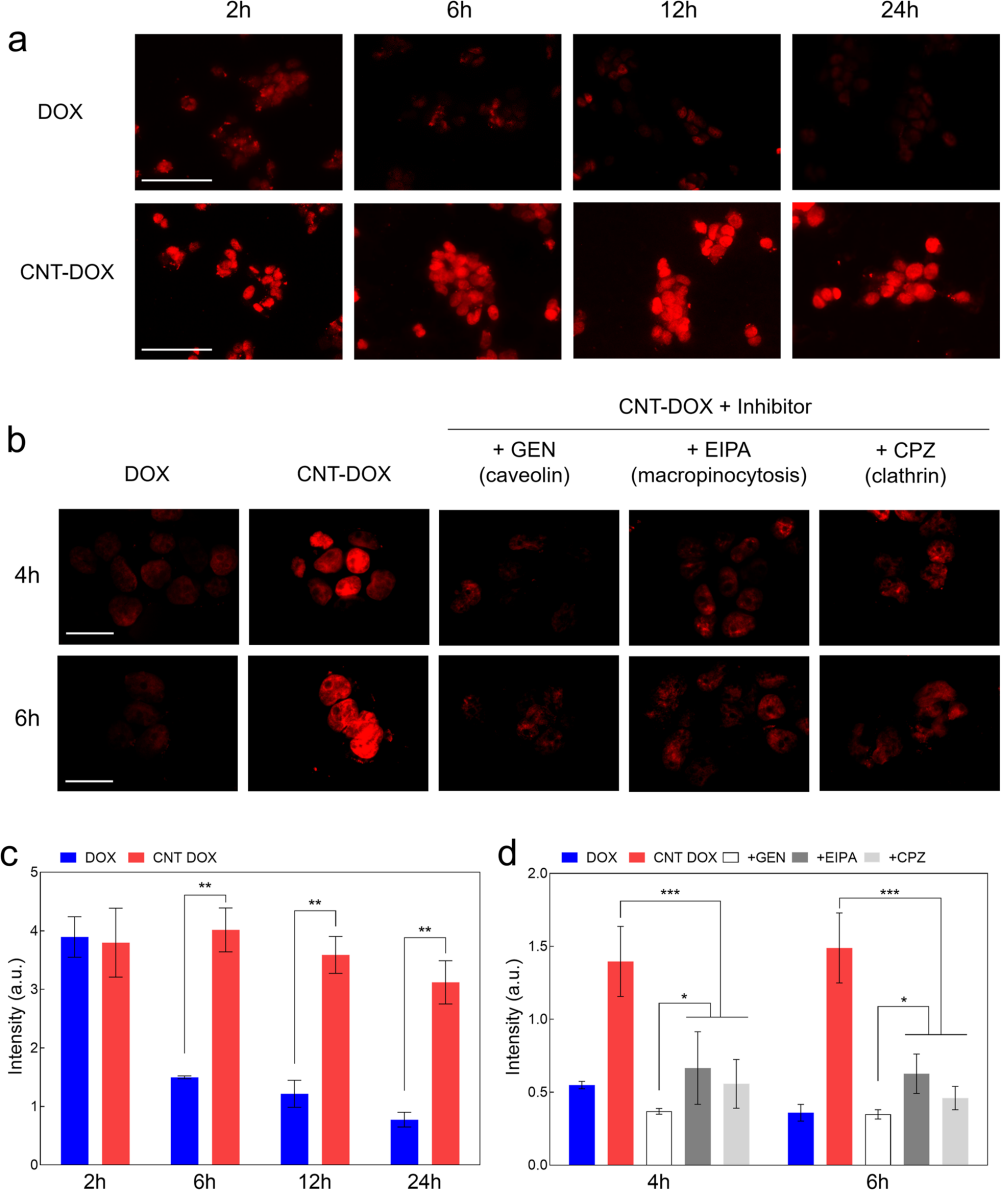
Uptake analysis. **a** Confocal images showing H69AR cells treated with the nanodrug. In H69AR cells, increased uptake of nanodrugs was confirmed. The scale bar is 75 μm. **b** Confocal images showing H69AR cells treated with the nanodrug and different types of uptake channel inhibitors were treated to examine uptake channels. All tested uptake pathways were identified (major: caveolae-mediated endocytosis, minor: macropinocytosis-mediated and clathrin-mediated endocytosis). The scale bar is 75 μm. **c** Florescence intensity (nuclear DOX uptake) analysis of H69AR cells treated with free DOX and the nanodrug. **d** Fluorescence intensity analysis of H69AR cells for comparison of major intracellular uptake pathways by the nanodrug. Caveolin channel was the most significant uptake channel compared to clathrin and macropinocytosis. Data are presented as the mean ± SEM (n = 6). *p < 0.05, **p < 0.01 and ***p < 0.001

Nanodrug uptake has been reported to occur by ATP-assisted endocytosis (i.e., dynamin dependent) when intracellular uptake occurs [33]. Representative uptake channels of nanodrug are clathrin-, caveolae-, and macropinocytosis-mediated endocytosis [34]. Sustained nanodrug internalization without drug efflux by resistant cancer cells is an important factor that can increase the efficacy of nanodrugs on drug-resistant cancer cells [30]. For investigating the nanodrug internalization and increasing the associated anticancer efficacy, the confocal analysis of H69AR cells was analyzed along with the treatment with CNT-DOX and DOX at different time points (Fig. 3a, b). In the DOX-treated group, a small DOX intensity was observed in the nucleus within 2 h; however, DOX was rapidly released from the nucleus after 2 h (Fig. 3a and c). The CNT-DOX-treated group showed a consistently high intensity of the DOX signal in the nucleus from 2 to 24 h (Fig. 3a and c). Normal lung cancer cells (A549), without resistance to DOX, showed that 60–100 nm nanodrugs increased overall anticancer efficacy against lung cancer cells (A549) through increased caveolin channel activity, confirming the therapeutic efficacy of 60–100 nm nanodrugs in vivo. [31] For DOX resistance cancer cells (H69AR), endocytosis inhibitors, such as chlorpromazine (CPZ), ethyl isopropyl amiloride (EIPA), and genistein (GEN), were used to treat lung cancer-resistant cells, to examine the internalization pathways of the nanodrug (Fig. 3b, c). Specifically, confocal analysis of the uptake and clearance over 24 h showed that the intracellular DOX signal of CNT-DOX was 4–5 times greater than that of free DOX (Fig. 3a–c). Among these, we confirmed that caveolin acts as a major uptake pathway (Fig. 3a–c). CNT-DOX with diameter of 60–100 nm penetrated drug-resistant tumor cells via three uptake pathways (Fig. 3b, c). In addition, CNT-DOX remained inside the cell for up to 24 h, unlike DOX, which was completely pumped out from the cell after 2 h (Fig. 3a–c). Interestingly, it was confirmed that CNT-DOX with a size of 10 nm showed slightly less intracellular uptake levels than CNT-DOX with a size of 60–100 nm (Additional file 1: Fig. S4a). Specifically, CNT-DOX with a diameter of 60–100 nm involved with three major intracellular uptake pathways (e.g., caveolin, clathrin, macropinocytosis), but caveolin was the most influential uptake pathway among them (Fig. 3b). Although calthrin appears to be the most influential uptake pathway among three major pathways for CNT-DOX with a diameter of 10 nm, those CNTs shows almost identical uptake levels (Additional file 1: Fig. S4b).

### Intracellular trafficking via EE and late endosome (LE) analysis

**Fig. 4:**
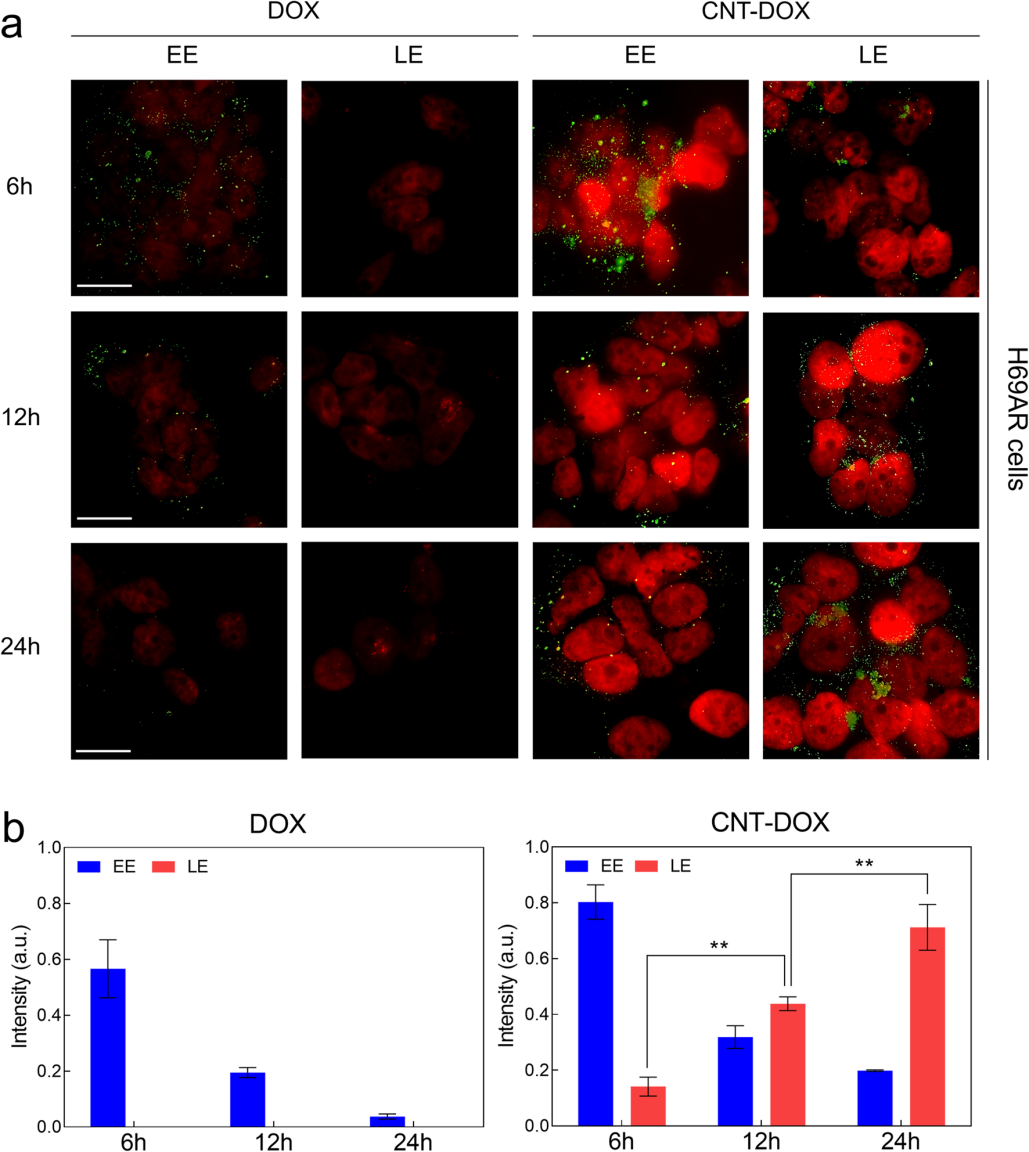
Intracellular trafficking of nanodrugs. **a** Confocal microscopy images visualizing DOX intensity (red) in the nuclei of H69AR cells and the indicated vesicles (early endosomes (EEs) and late endosomes (LEs), green) after treatment with free DOX and the CNT-DOX for 6, 12 and 24 h. The scale bar is 75 μm. **b** Time-dependent fluorescence intensity of EE and LE after treatment with free DOX and CNT-DOX for the indicated times (6, 12, and 24 h). The fluorescence intensities of LE were only found after incubating with CNT-DOX for 6, 12, and 24 h and analyzed by normalizing the fluorescence intensities of confocal images. All data represent the mean ± SEM (n = 6). *p < 0.05, **p < 0.01 and ***p < 0.001

The literature has shown that covalent conjugation of CNT-DOX in intracellular trafficking assays represents a more stable drug conjugation style in intracellular drug delivery systems [35]. In this study, intracellular drug delivery analysis showed that early endosome (EE) and LE signal intensities were analyzed in normal lung cancer cells (A549 and H446 cells) and multidrug-resistant SCLC cells (H69AR cells) (Fig. 4 and Additional file 1: Fig. S5). Confocal analysis confirmed that the EE intensity was greater for CNT-DOX than for DOX after 6 h (Fig. 4a, b and Additional file 1: Fig. S5a, b). However, the EE signal gradually decreased for DOX and CNT-DOX (Fig. 4a, b and Additional file 1: Fig. S5a, b). In multidrug-resistant SCLC cells (H69AR), the initial endosomal intensity of DOX almost disappeared 24 h after treatment. However, we confirmed that EE was maintained after 24 h in the CNT-DOX treatment group (Fig. 4a, b). In addition, the late endosomal (LE) intensity in H69AR gradually increased after 6 h, showing the strongest intensity after 24 h (Fig. 4a, b). Specifically, the LE intensity of DOX in multidrug-resistant small-cell lung cancer cell lines could not be observed after 24 h of drug treatment. In the CNT-DOX-treated group, LE appeared after 6 h, and strong LE formation was observed after 24 h (Fig. 4a, b). The obtained results clearly indicate that the formation of LE vesicles in H69AR was only observed on CNT-DOX. [30]

### Intracellular acidic pH analysis

**Fig. 5:**
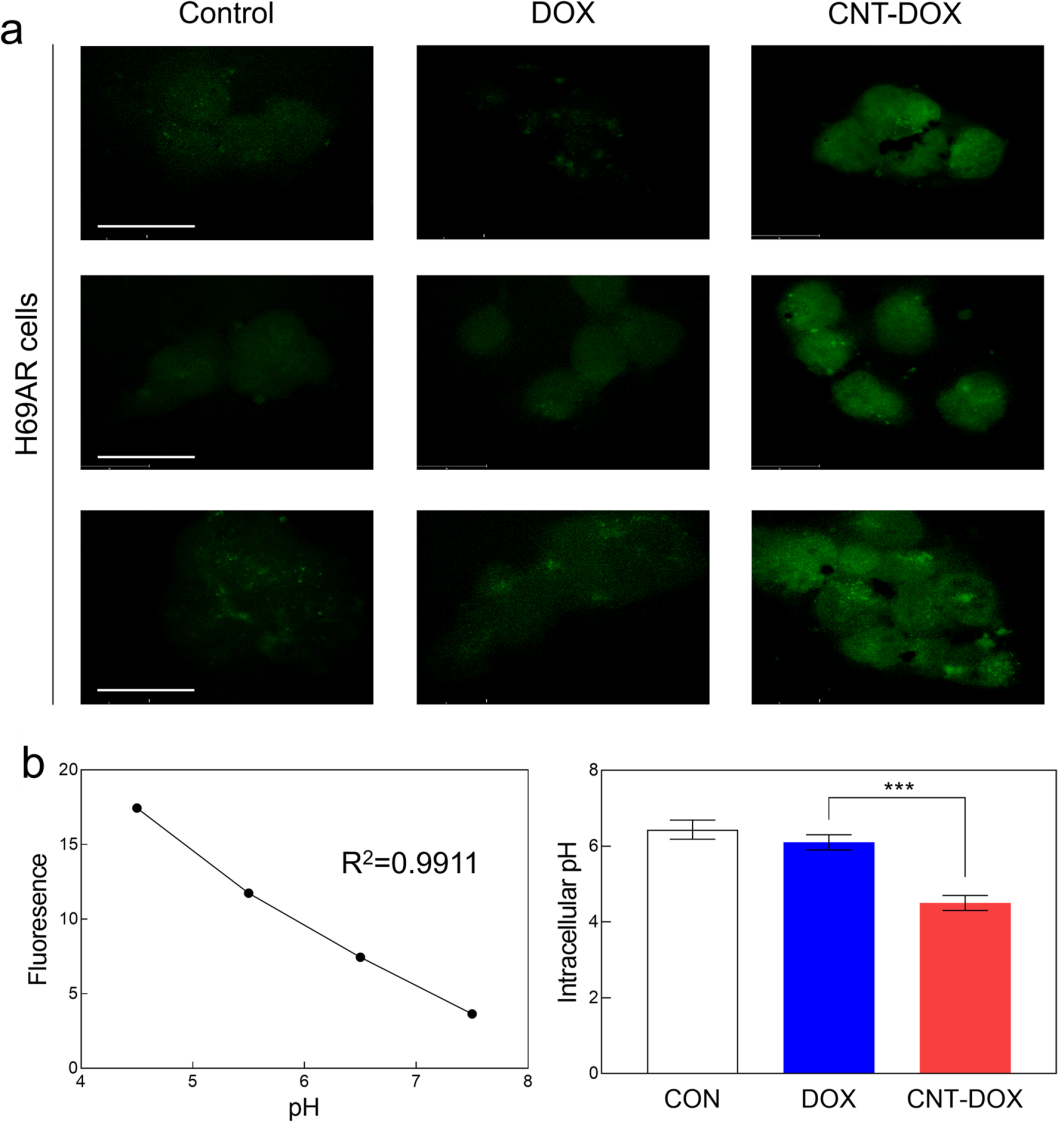
Intracellular pH analysis. **a** Confocal images showing the pH Rodo staining in H69AR cells treated with DOX or CNT-DOX for 24 h. H69AR cells treated with CNT-DOX showed a more acidic condition than the DOX-treated group. The scale bar is 75 μm. **b** Calibration curve graph for intracellular pH measurement in H69AR cells, and bar graph showing intracellular pH level. All data represent the mean ± SEM (n = 6). ***p < 0.001

Changes in intracellular pH are markers of acidic cancer cells. In this study, environmental changes in intracellular pH were analyzed in A549, H446, and H69AR cells. Examination of efflux pumps of multidrug-resistant cells revealed clear differences in pH changes due to CNT-DOX compared with DOX (Fig. 5 and Additional file 1: Fig. S6). As was confirmed in the confocal images, the pH inside normal lung cancer cells was maintained between 6.2 and 6.5 for all three tumor cells (Fig. 5 and Additional file 1: Fig. S6). However, the intracellular pH of lung cancer cells decreased to below 5 after CNT-DOX treatment (Additional file 1: Fig. S6a, b). It was also maintained at approximately pH 6 in multidrug-resistant SCLC cell lines without drug treatment (Fig. 5a, b). However, the pH inside the multidrug-resistant small cells after CNT-DOX administration was significantly lowered to an average pH of 4.5 (Fig. 5). This may be indirect evidence that CNT-DOX induced apoptosis of multidrug-resistant cancer cells in an acidic intracellular environment. [36, 37] In summary, we confirmed that CNT-DOX uptake into multidrug-resistant cells through a specific endocytosis pathway evaded drug efflux by mrp-1 and lowered the intracellular pH of cancer cells. Specifically, the prepared nanodrugs induced changes in the charge of intracellular organelles in an acidic environment in multidrug-resistant cancer cells, which could induce more apoptosis (Fig. 5).

### Cancer apoptosis analysis by nanodrugs

**Fig. 6:**
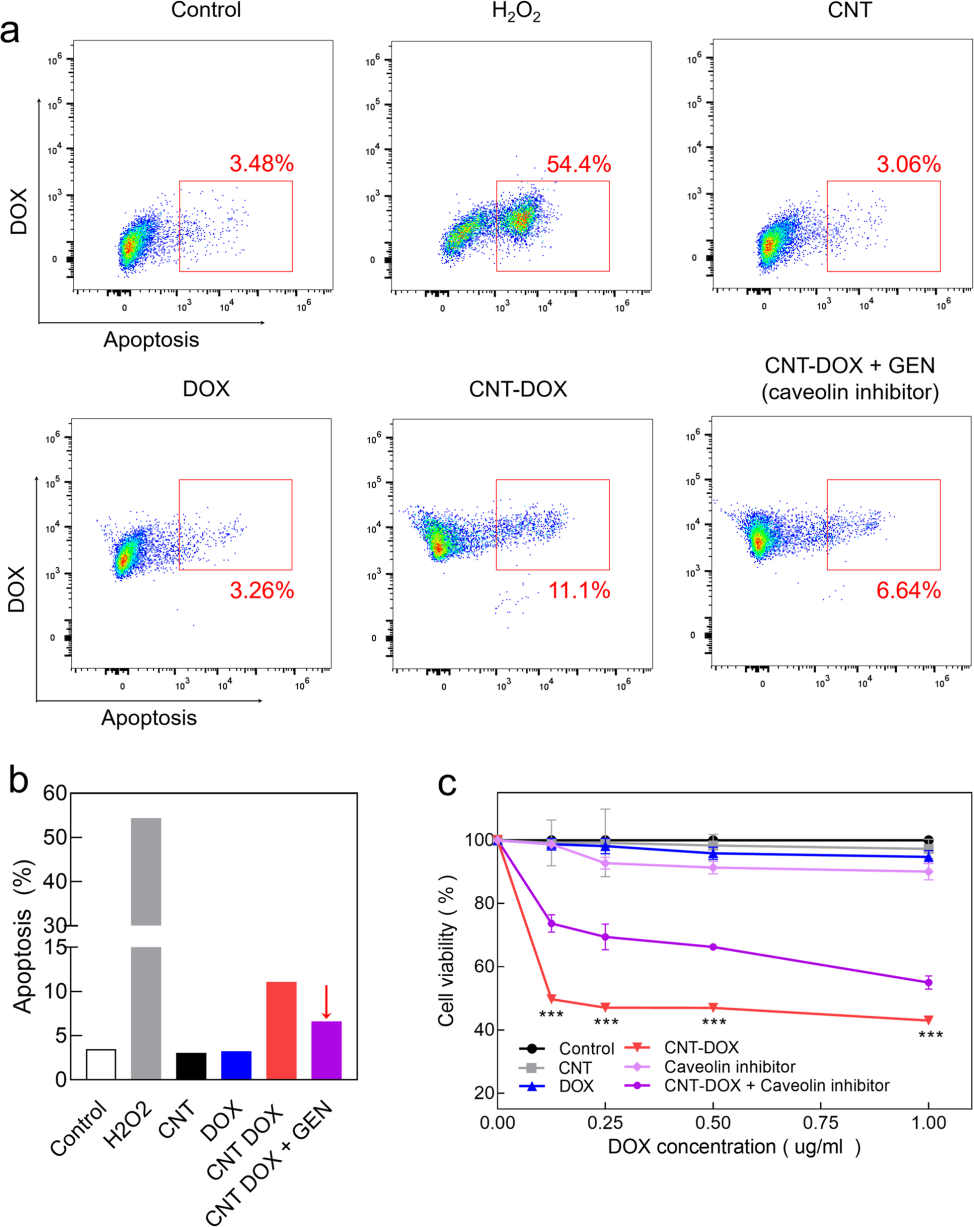
Apoptosis (Annexin V) and cell viability (MTT) analysis. **a** Apoptosis analysis using a fluorescence-activated cell sorting (FACS) assay after treatment with CNT, DOX, CNT-DOX, and CNT-DOX with GEN (caveolin endocytosis inhibitor) for 24 h. As a positive control, hydrogen peroxide (H2O2) was used. Data shows significant apoptosis on CNT-DOX compared with DOX. **b** Bar graphs illustrating apoptosis levels quantified by Annexin in H69AR cells after nanodrug treatment. **c** Cell viability analysis using an MTT assay after treatment with CNT, DOX, CNT-DOX, Caveolin inhibitor (GEN), and CNT-DOX with GEN for 48. Data shows significant decrease of cell population on CNT-DOX but recovered after treatment of caveolin inhibitor. All data represent the mean ± SEM (n = 10). ***p < 0.001

We investigated the apoptosis of CNT-DOX in multidrug-resistant tumor cells (Fig. 6a–c). H69AR cells were treated with CNT-DOX and DOX, and after 24 h, stained with Annexin V Pacific Blue. Apoptosis was confirmed by FACS (Fig. 6a, b). FACS analysis showed that DOX had a negligible effect on the apoptosis of multidrug-resistant tumor cells. However, CNT-DOX showed four times more apoptotic FACS signals in H69AR cells than DOX did (Fig. 6a, b). Notably, we confirmed that the apoptotic effect was halved after the blocking of caveolin channels after treatment with genistein, a caveolin endocytosis pathway inhibitor (Fig. 6a, b). This demonstrates the critical role of caveolin uptake channels in multidrug-resistant SCLC cells, as identified in the aforementioned results (Fig. 3). Similarly, apoptosis analysis using FACS showed increased anticancer efficacy in multidrug-resistant tumor cells (Fig. 6a, b). In addition, cell viability results using MTT assay showed that that of the DOX-treated group was as high as 98%, similar to that of the control group in H69AR cells. By contrast, the CNT-DOX-treated group showed that the cell viability was below 50%, which shows that CNT-DOX has a significant impact on multidrug-resistant tumor cells (Fig. 6c). On the other hand, the CNT-DOX treated group with a length of 10 nm showed about 75% cell viability, confirming that the anticancer efficacy was significantly lower than that of CNT-DOX with a length of 60–100 nm (Additional file 1: Fig. S7). Similar to the FACS results, the group treated with caveolin endocytosis inhibitors with CNT-DOX showed a slight recovery of the H69AR survival rate, approximately 60%-70% (Fig. 6c). However, in the group treated with caveolin inhibitor alone, the survival rate of H69AR was more than 90% similar to that of DOX. Therefore, the caveolin inhibitor did not significantly affect apoptosis (Fig. 6c and Additional file 1: Fig. S8a). Overall, this is due to the drug defense mechanism that releases DOX to the outside of the cell via mrp-1 activity in multidrug-resistant tumor cells. This occurs because of a drug defense mechanism that releases DOX to the outside of the cell due to mrp-1 activity in multidrug-resistant tumor cells, and we demonstrated that free DOX alone does not induce apoptosis in multidrug-resistant tumor cells.

### Mitochondrial damage and associated ATP reduction of H69AR

**Fig. 7:**
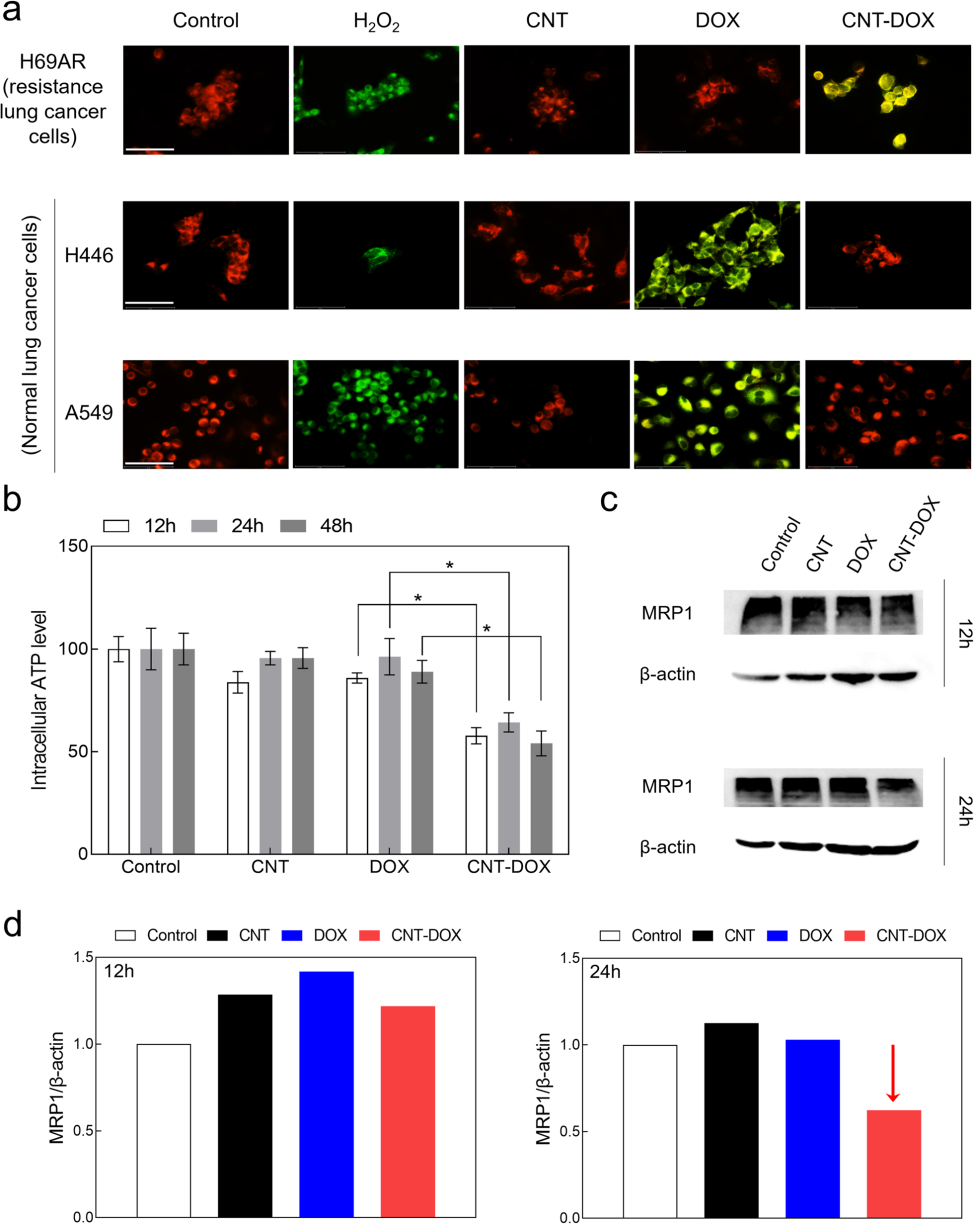
Changes in mitochondrial membrane potential (ΔΨ_m_) and reduction of efflux pump. **a** Confocal images of H69AR showing the JC-1 staining that the depolarized mitochondria (J-monomer, green) were only observed on CNT-DOX treated group and polarized mitochondria (J-aggregate, red) membrane potentials were found on DOX after 24 h. As a positive control, hydrogen peroxide (H2O2) was used. Specifically, CNT-DOX significantly influenced mitochondrial membrane potential in multidrug-resistant lung cancer cells (i.e., H69AR), whereas normal lung cancer cells (i.e., A549 and H446) did not show any notable changes in membrane potential. The scale bar is 75 μm. **b** Relative intracellular ATP levels in H69AR cells treated with CNT, DOX, and CNT-DOX for the indicated times (12, 24, and 48 h). **c** Western blot analysis of mrp-1 expression in H69AR cells treated with the nanodrugs (12 and 24 h). **d** Bar graphs illustrating mrp-1 protein levels quantified by western blot in H69AR cells after drug treatment. Decrease of mrp-1 was only observed on CNT-DOX treated group after 24 h. All protein levels were normalized to the b-actin protein level. All data represent the mean ± SEM (n = 6). *p < 0.05

In the literature, selecting the appropriate type of nanodrug could increase the probability of drug accumulation in the mitochondria, because drugs can target mitochondria, which induce apoptosis by disrupting the energy-generating sub-organs in cancer cells [30]. The literature has identified that the PEG coat of a nanodrug is advantageous for drug delivery to the mitochondria, and covalent bonding is highly effective for drug delivery to the nucleus [30]. Because mitochondria are the most organelles for ATP production, selective mitochondrial targeting of nanodrugs was confirmed by investigating changes in mitochondrial membrane potential (JC-1) (Fig. 7a). Notably, in A549 and H446 cells, which are normal tumor cells, we confirmed that the CNT-DOX-treated group was delivered directly to the nucleus without passing through the mitochondria (Fig. 7a). By contrast, H69AR, a multidrug-resistant cell line, significantly increased the change in mitochondrial membrane potential (Fig. 7a). We interpreted this as resulting in the accumulation of the released drug from the EE to the LE and into the mitochondria (Fig. 7a). In addition, CNT-DOX delivered to H69AR created a more acidic environment inside the multidrug-resistant tumor cells, causing electric changes and disruption of intracellular organelles.

Analysis of mrp-1 and ATP, which are related to efflux pumps of cancer-resistant cells, is a critical factor for understanding efflux in multidrug-resistant tumor cells [38]. First, to confirm the change in ATP, the energy source of mrp-1, the intracellular ATP change was confirmed after 12 h, 24 h, and 48 h after drug treatment in H69AR cells (Fig. 7b). In the group treated with CNT and DOX alone, there was no significant difference in ATP from 12 to 48 h; by contrast, in the CNT-DOX treatment group, the ATP level was greatly reduced by half after 12 h (Fig. 7b). Because ATP is the main energy source for mrp-1, a decrease in intracellular ATP may activate mrp-1 expression. In addition, rapid intracellular ATP consumption over a short period may induce cell apoptosis [39]. Thus, the decrease in ATP by CNT-DOX suggests a significant possibility of efflux malfunction in multidrug-resistant tumor cells.

Decreased mrp-1 expression due to ATP reduction was confirmed in multidrug-resistant tumor cells (Fig. 7c, d). First, the overexpression of mrp-1 in H69AR cells was confirmed by a comparison with normal cancer cells (Additional file 1: Fig. S8b), and the change in mrp-1 expression at 12 h and 24 h after drug treatment was confirmed by western blotting (Fig. 7c, d). Western blot results confirmed that the expression of mrp-1 was higher in the group treated with CNT and DOX than in the control group and was maintained at 24 h (Fig. 7c, d). On the other hand, in the CNT-DOX-treated group, the expression of mrp-1 was slightly higher after 12 h but decreased after 24 h compared with the control group (Fig. 7c, d). The full-length staining of western blot analysis is presented in the supplementary information (Additional file 1: Fig. S9). In summary, the decrease of intracellular ATP level by mitochondrial damage was evident after 12 h of drug administration. As such, the decreased ATP energy level within 12 h may influence not greatly subsequent the expression of mrp-1 (consuming ATP as a main energy source). However, it was confirmed that the expression of mrp-1 decreased after 24 h and this represent that sustained lower ATP significantly influenced mrp-1 protein synthesis (Fig. 7).

### In vivo analysis

**Fig. 8:**
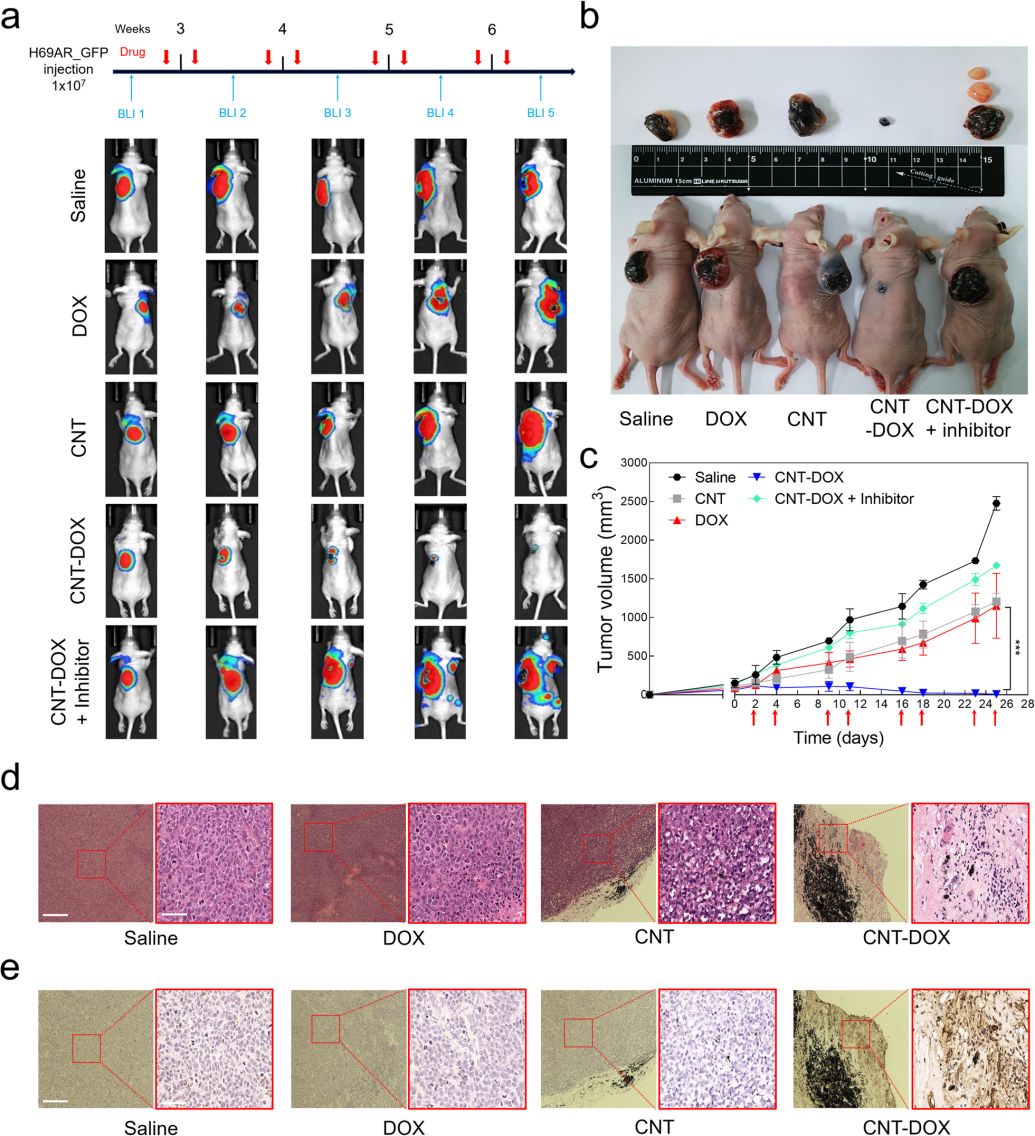
Antitumor efficacy of nanodrug on xenograft model mice. **a** BLI of luciferase expression in H69AR_**flu-GFP**_ tumor-bearing mice after treatment with PBS, DOX (5 mg/kg), CNT (5 mg/kg), CNT-DOX (5 mg/kg), and CNT-DOX with caveolin inhibitor (5 mg/kg). Red arrow indicates the day of drug injection. **b** Photographs of H69AR tumor-bearing mice and tumor tissues in week four after drug injection. The smallest tumors with the CNT-DOX treatment are indicated by red arrows. **c** After the tumor size reached 100 mm^3^, drugs were injected twice per week for 4 weeks, and tumor size was measured. The therapeutic effect of CNT-DOX on a xenograft mouse model is shown. **d** Representative images of Hematoxylin and eosin (H&E) staining from H69AR tumor tissues treated with PBS, DOX (5 mg/kg), CNT (5 mg/kg), and CNT-DOX (5 mg/kg). Scale bar of the low- and high-resolution image is 275 μm and 75 μm, respectively. **e** Terminal deoxynucleotidyl transferase dUTP nick end labeling (TUNEL) staining from H69AR tumor tissues clearly exhibited greater apoptosis on CNT-DOX than on the other groups. Scale bar of the low- and high-resolution image is 275 μm and 75 μm. All data are presented as mean ± SEM (n = 5). ***p < 0.001

Mice were injected with H69AR_Fluc-GFP_ (1 × 10^7^ in 100 ul of PBS) between the dorsal epithelium and endothelium, and the size of the cancer was confirmed by measuring fluorescence through IVIS once per week (Fig. 8a). When the size of the tumor was greater than 100 mm^3^, free DOX and 60–100 nm CNTs, CNTs covalently conjugated with DOX (CNT-DOX), and simultaneously synthesized nanodrugs and inhibitors of the caveolin uptake channel (CNT-DOX + inhibitor) were directly injected into the tumors of mice. Drug injections were performed twice per week, and BLI was performed once per week to analyze changes in luciferase intensity and the size of lung cancer tumors (Fig. 8a). After four injections of the drug, a significant decrease in luciferase intensity was observed in the tumors of the CNT-DOX-treated group, whereas the PBS, DOX, CNT, and CNT-DOX + inhibitor groups did not inhibit tumor growth (Fig. 8a). Notably, we confirmed that the CNT-DOX + caveolin inhibitor group not only interfered with tumor suppression but also caused tumor metastasis (Fig. 8a, b). This proves that caveolin plays an important role in the uptake pathway in tumor cells in the 60–100 nm CNT-based nanodrugs, as demonstrated in another study. During eight injections, the changed tumor size showed values consistent with the BLI results (Fig. 8b, c). The tumors of the PBS, DOX, CNT, and CNT-DOX + inhibitor groups grew more than 10 times their initial tumor size because of the rapid growth rate throughout the administration period, whereas the CNT-DOX group was confirmed to be so small that almost no tumors remained (Fig. 8b, c). After sacrificing the mice, H&E staining of the tumor tissue obtained from each group identified many regions of the tumor tissue and, importantly, confirmed the cell density of tumor cells within the tumor tissue (Fig. 8c). Notably, the tumor tissue of the CNT-DOX group showed morphological collapse due to a decrease in the density of the cell nuclei (Fig. 8c). In addition, the transferase dUTP nick end labeling assay results of the tumor tissue of each group showed the degree of apoptosis in the tumor (Fig. 8d). The apoptosis signal did not appear in tumor tissues of the Saline, DOX, CNT, and CNT-DOX + inhibitor groups, but we confirmed that the apoptosis signal was strongly displayed in the tumor tissues of the CNT-DOX group (Fig. 8e and Additional file 1: Fig. S10). The results demonstrate that CNT-DOX of a specific size (60–100 nm) has the advantage of intracellular uptake through caveolin and can effectively eliminate multidrug-resistant tumors.

### Toxicity analysis

**Fig. 9:**
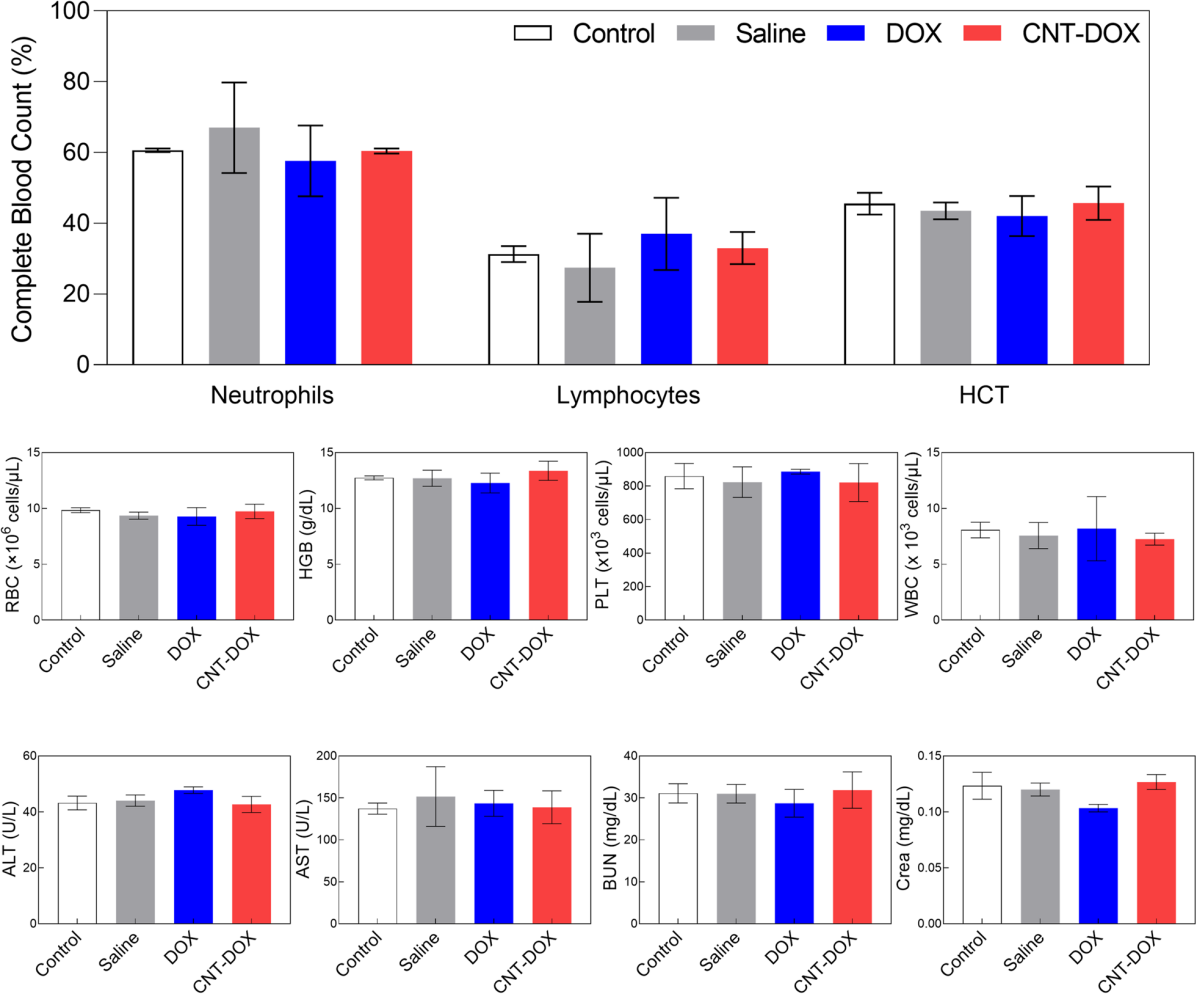
Toxicity evaluation by nanodrug on xenograft model mice. BALB/c nude mice were sacrificed 4 weeks (BLI 5) after H69AR injection and showed no notable changes in general immunotoxicity levels in CNT-DOX-treated mice compared with the control group. *HCT* hematocrit, *RBC* red blood cells, *HGB* hemoglobin, *PLT* platelets, *WBC* white blood cells, *ALT* abbreviations enzymatic activity of alanine transaminase, *AST* enzymatic activity of aspartate aminotransferase, *BUN* blood urea nitrogen. All data are presented as the mean ± SEM (n = 5)

In vivo safety is an important factor in the preclinical evaluation of groups of nanomaterial-based drugs. In this study, because the drug was administered intratumorally, we hypothesized that the nanodrugs would not have a significant effect on in vivo toxicity. However, the in vivo toxicity of CNT-based nanodrugs has been a very sensitive issue. In this study, in vivo blood was tested for toxicity in all administered drug groups (Fig. 9). Peripheral circulating leukocyte counts (WBC, lymphocytes, and neutrophils) and representative hematologic markers (red blood cells, hemoglobin, and PLT) showed no change in any group compared with control mice (Fig. 9). Aspartate transaminase (AST) and alanine aminotransferase (ALT), which are representative serum biochemical markers of hepatotoxicity, were identified and compared with those in control mice; they were confirmed to be within the normal range in all groups (Fig. 9). In addition, in the analysis of blood urea nitrogen (BUN) and creatinine (Crea), which are representative indicators of renal function, results within the normal range were confirmed in all groups compared with control mice (Fig. 9). Again, this means that CNT-DOX and DOX used to treat multidrug-resistant tumors did not induce appreciable immunotoxicity within the administered dose.

## Conclusion

For SCLC, chemotherapy or radiation therapy has mainly been used instead of surgery. [4, 5] However, continuous exposure to chemotherapy has created resistance to a wide range of anticancer drugs. Despite significant advances in chemotherapy, the rapid emergence of drug resistance limits the benefits of SCLC treatment, leading to low survival rates and prognoses [1]. In the literature, a positive anticancer effect was confirmed in lung cancer on the basis that CNTs can accumulate in the lungs [40]. Furthermore, the selection of nano-conjugation styles (i.e., amide covalent bond, PEG coat, and encapsulation) enables selective intracellular targeting and ultimately increases cytotoxicity in cancer [41].

In this study, CNT-based nano cancer drugs of a specific size could evade the efflux pump mechanism of multidrug-resistant small-cell lung cancer cells and effectively treat tumors. Specifically, we confirmed that CNT-DOX, a nanodrug covalently bound to H69AR, a SCLC cell resistant to DOX, effectively killed multidrug-resistant tumors. Resistant cells had absorbed DOX, but the assimilated drug was rapidly released and remained inside the cell. By contrast, CNT-DOX remained inside the cell even at low concentrations, which confirmed that the uptake of the drug into the cell by caveolin-dependent endocytosis was delivered to the nucleus through the EE and LE pathways. In addition, CNT-DOX delivered to multidrug-resistant tumor cells created a more acidic environment inside the multidrug-resistant tumor cells, causing electric changes and disrupting intracellular organelles. Consequently, changes in the acidic intracellular microenvironment of multidrug-resistant cancer cells induced by CNT-DOX led to mitochondrial damage. Ultimately, mitochondrial damage in multidrug-resistant cancer cells inhibits the production of ATP, an essential energy source, blocking the activation of mrp-1 and inducing apoptosis. It has been suggested that CNT-based nanodrugs of a specific size could overcome drug resistance and induce apoptosis by changing the intracellular microenvironment and destroying intracellular organelles. In conclusion, the specific size of the nanodrug created greater apoptosis, enhanced therapeutics for multidrug-resistant cancer cells, and provided insights into a strategy that can overcome multidrug-resistant lung cancer cells.

## Supplementary Information

**Additional file 1: Fig. S1.** The material properties of CNT-DOX. **Fig. S2.** TEM analysis of pure CNT and oxidized CNT. (a) TEM image of pure and (b) oxidized CNTs show the difference in the surface structures on the surface. Scale bar shows 20 nm. **Fig. S3.** Photoluminescence (PL) analysis of CNT-DOX. Comparison of the luminescence quenching ratios of DOX, CNT+DOX (mix), and CNT-DOX (covalent conjugation) show that the evidence of strong covalent bonding (amide bonds) between DOX and CNT. **Fig. S4.** Intracellular uptake comparison of CNT-DOX (10 nm) and CNT-DOX (60-100 nm). (a) Confocal microscopy images visualizing DOX intensity (red) in the nuclei of CNT-DOX (10 nm and 60-100 nm). (b) Confocal images showing H69AR cells treated with the CNT-DOX (10 nm) and different types of uptake channel inhibitors were treated to examine relative activation of uptake channels. The scale bar shows 75 μm. **Fig. S5.** Intracellular trafficking in normal lung cancer cells. Confocal microscopy images and calibration bar graph visualizing DOX intensity (red) in the nuclei of (a) non-small cell lung cancer cells (A549 cells) and (b) small cell lung cancer cells (H446 cells), and the indicated vesicles (early endosomes (EEs) and late endosomes (LEs), green) after treatment with free DOX and CNT-DOX after 6, 12, and 24 h. Scale bar, 75 μm. Data represent the mean ± SEM (n = 6). **Fig. S6.** Intracellular pH analysis in normal lung cancer cells. Confocal images and calibration curve graph showing Rodo staining in (a) A549 cells and (b) H446 cells treated with DOX or CNT-DOX for 24 h. A549 and H446 cells treated with CNT-DOX showed more acidic conditions than those in the DOX-treated group. Scale bar, 75 μm. Data represent the mean ± SEM (n = 6). **p < 0.01 and ***p < 0.001. **Fig. S7.** Viability analysis. Cell viability analysis using an MTT assay after treatment with CNT (10 nm), DOX, CNT-DOX (both 10 nm and 60-100 nm). CNT-DOX (60-100 nm) shows selective anticancer efficacy compared with other tested drugs (both CNT-DOX (10 nm) and DOX). **Fig. S8.** Apoptosis and western blot analysis. (a) Apoptosis analysis using a fluorescence-activated cell sorting (FACS) assay after treatment with GEN (caveolin endocytosis inhibitor) for 24 h. (c) Western blot analysis of mrp-1 expression in multidrug-resistant lung cancer cells (H69AR cells) and normal lung cancer cells (A549 and H446 cells). **Fig. S9.** The full-length staining of western blot analysis. The full-length western blot analysis of (a) Fig. 7c and (b) Supplementary Fig. 3b. **Fig. S10.** Antitumor efficacy of xenograft mouse model. Terminal deoxynucleotidyl transferase dUTP nick-end labeling (TUNEL) staining of H69AR tumor tissues after treatment with CNT-DOX and GEN (caveolin endocytosis inhibitor). Far-field (left) and near-field (right) images shows of apoptosis (brown colors in immunohistochemical staining).
