# Supplementary material for: Overcoming multidrug-resistant lung cancer by mitochondrial-associated ATP inhibition using nanodrugs
Source: J Nanobiotechnology. 2023 Jan 12;21:12. doi: 10.1186/s12951-023-01768-8 (PMC9835376; doi:10.1186/s12951-023-01768-8)
Supplement: Supplementary file 1 — Additional file 1: Fig. S1. The material properties of CNT-DOX. Fig. S2. TEM analysis of pure CNT and oxidized CNT. (a) TEM image of pure and (b) oxidized CNTs show the difference in the surface structures on the surface. Scale bar shows 20 nm. Fig. S3. Photoluminescence (PL) analysis of CNT-DOX. Comparison of the luminescence quenching ratios of DOX, CNT+DOX (mix), and CNT-DOX (covalent conjugation) show that the evidence of strong covalent bonding (amide bonds) between DOX and CNT. Fig. S4. Intracellular uptake comparison of CNT-DOX (10 nm) and CNT-DOX (60-100 nm). (a) Confocal microscopy images visualizing DOX intensity (red) in the nuclei of CNT-DOX (10 nm and 60-100 nm). (b) Confocal images showing H69AR cells treated with the CNT-DOX (10 nm) and different types of uptake channel inhibitors were treated to examine relative activation of uptake channels. The scale bar shows 75 μm. Fig. S5. Intracellular trafficking in normal lung cancer cells. Confocal microscopy images and calibration bar graph visualizing DOX intensity (red) in the nuclei of (a) non-small cell lung cancer cells (A549 cells) and (b) small cell lung cancer cells (H446 cells), and the indicated vesicles (early endosomes (EEs) and late endosomes (LEs), green) after treatment with free DOX and CNT-DOX after 6, 12, and 24 h. Scale bar, 75 μm. Data represent the mean ± SEM (n = 6). Fig. S6. Intracellular pH analysis in normal lung cancer cells. Confocal images and calibration curve graph showing Rodo staining in (a) A549 cells and (b) H446 cells treated with DOX or CNT-DOX for 24 h. A549 and H446 cells treated with CNT-DOX showed more acidic conditions than those in the DOX-treated group. Scale bar, 75 μm. Data represent the mean ± SEM (n = 6). **p < 0.01 and ***p < 0.001. Fig. S7. Viability analysis. Cell viability analysis using an MTT assay after treatment with CNT (10 nm), DOX, CNT-DOX (both 10 nm and 60-100 nm). CNT-DOX (60-100 nm) shows selective anticancer efficacy compared with other tested d [file 12951_2023_1768_MOESM1_ESM.docx]

Supplementary information for

**Destroying Multidrug-Resistant Lung Cancer by Mitochondrial-Associated ATP Inhibition using Nanodrugs**

Jun-Young Park^1,2,*^, Gyu-Ho Lee^3,*^, Kwai Han Yoo^4,†^ and Dongwoo Khang^1,2,3,†^

*^1^Lee Gil Ya Cancer and Diabetes Institute, Gachon University Incheon 21999, South Korea*

*^2^Department of Health Sciences and technology, GAIHST, Gachon University, Incheon 21999, South of Korea*

*^3^Department of Physiology, College of Medicine, Gachon University, Incheon 21999, South Korea*

*^4^Department of Internal Medicine, Gachon University Gil Medical Center, College of Medicine, Incheon, 21565, South Korea*

^*^Equally contributed

^†^ E-mail: [dkhang@gachon.ac.kr](mailto:dkhang@gachon.ac.kr) or [khyoo@gilhospital.com](mailto:khyoo@gilhospital.com)


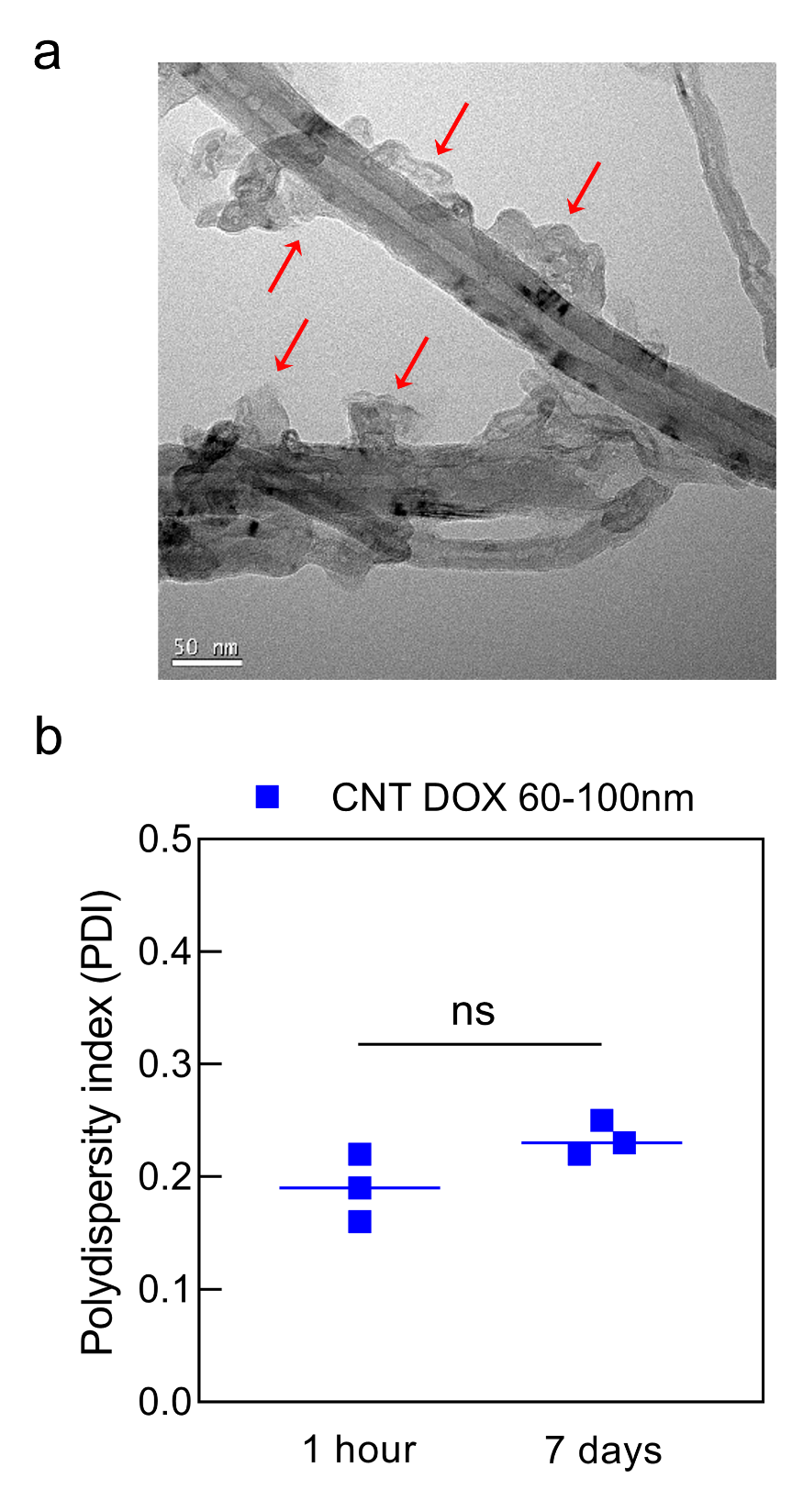


**Supplementary Fig. 1 The material properties of CNT-DOX**

(a) Far-field TEM image of CNT-DOX. Red arrows indicate covalently attached DOX surrounding on CNT. (b) Analysis of the polydispersity indexes of CNT-DOX (60-100 nm). in PBS (pH 7.4) shows that CNT-DOX keeps sufficient polydispersity in PBS after 7 days.


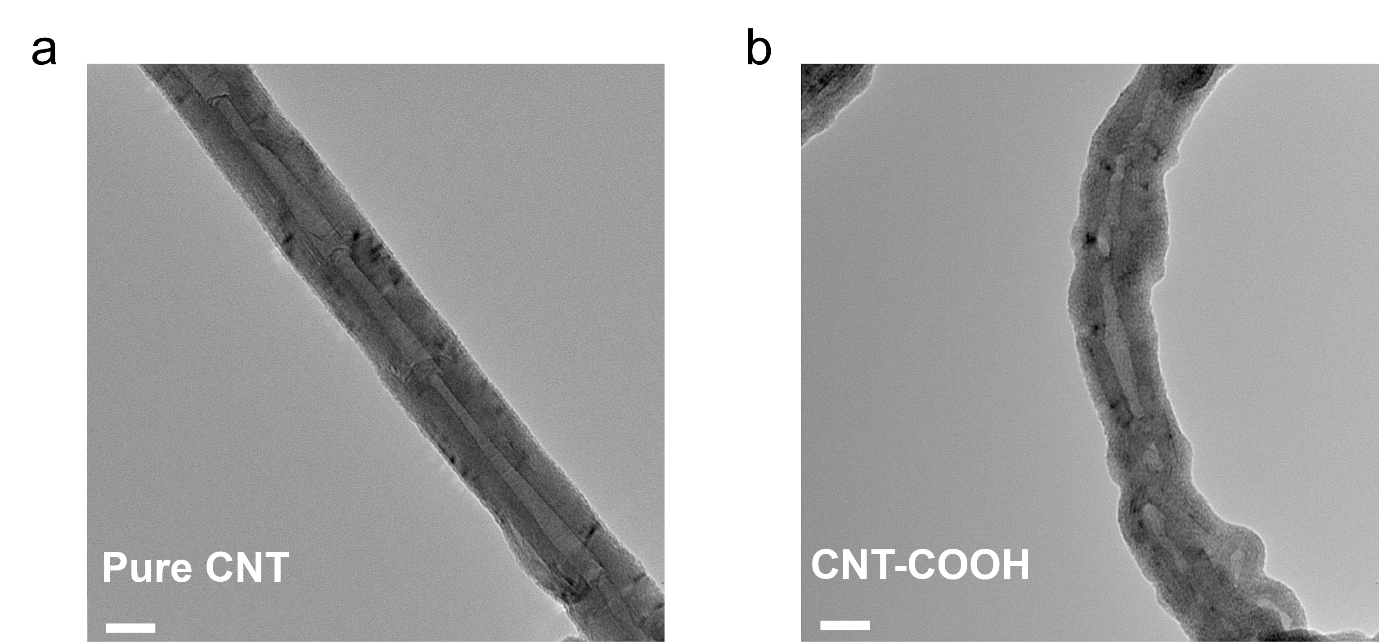


**Supplementary Fig. 2 TEM analysis of pure CNT and oxidized CNT**

(a) TEM image of pure and (b) oxidized CNTs show the difference in the surface structures on the surface. Scale bar shows 20 nm.


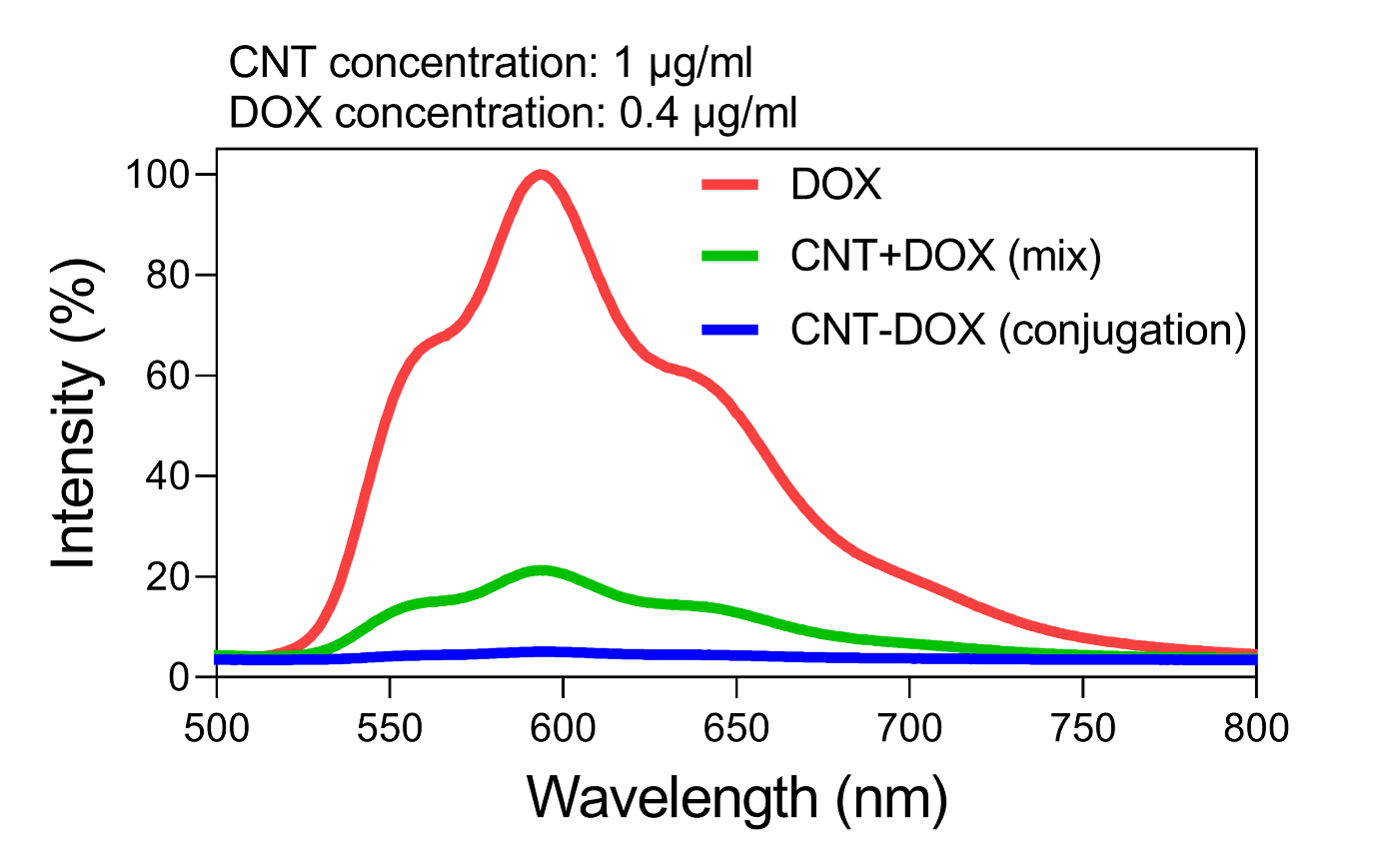


**Supplementary Fig. 3 Photoluminescence (PL) analysis of CNT-DOX**

Comparison of the luminescence quenching ratios of DOX, CNT+DOX (mix), and CNT-DOX (covalent conjugation) show that the evidence of strong covalent bonding (amide bonds) between DOX and CNT.


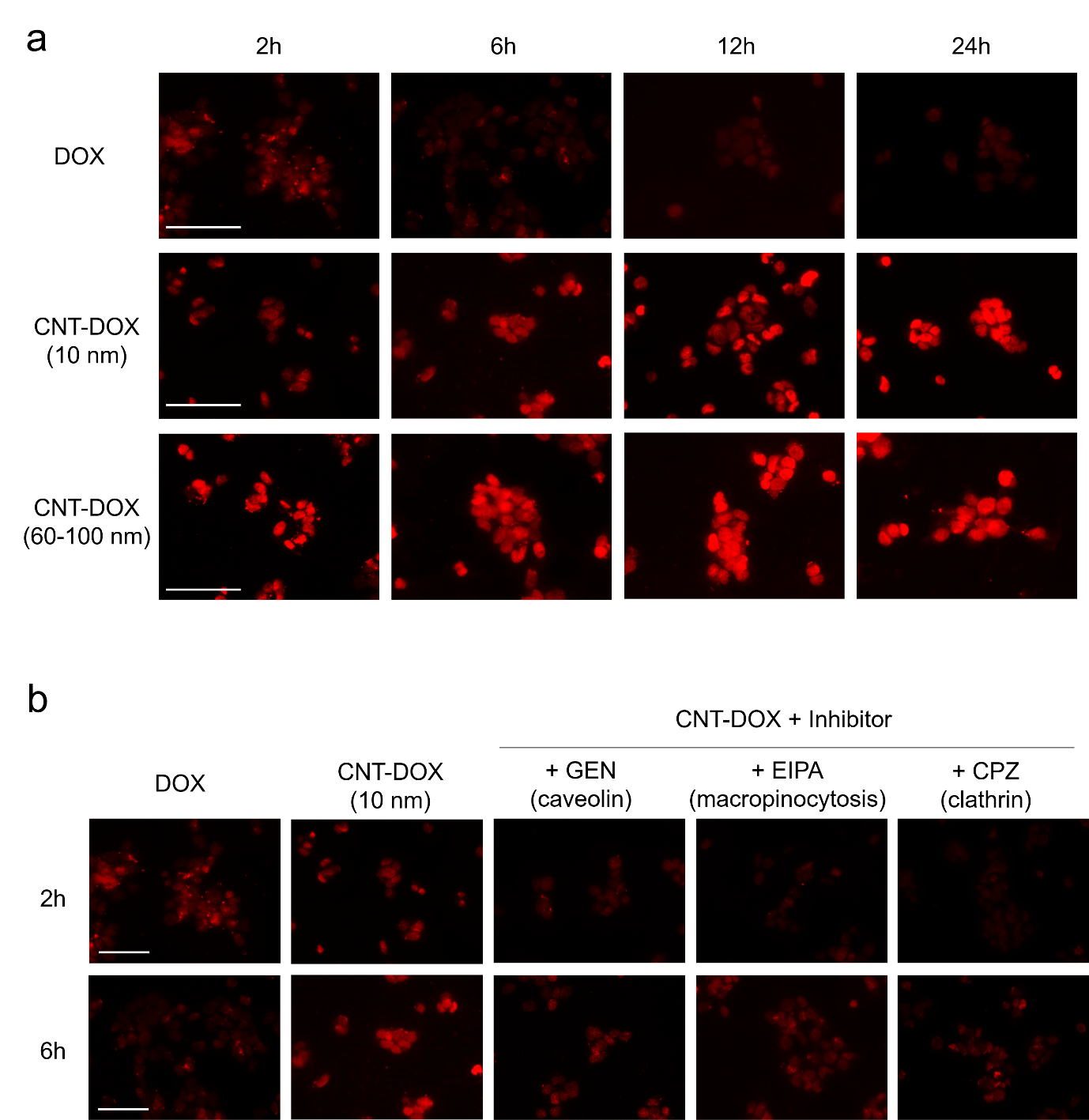


**Supplementary Fig. 4** **Intracellular uptake comparison of CNT-DOX (10 nm) and CNT-DOX (60-100 nm)**

(a) Confocal microscopy images visualizing DOX intensity (red) in the nuclei of CNT-DOX (10 nm and 60-100 nm). (b) Confocal images showing H69AR cells treated with the CNT-DOX (10 nm) and different types of uptake channel inhibitors were treated to examine relative activation of uptake channels. The scale bar shows 75 μm.


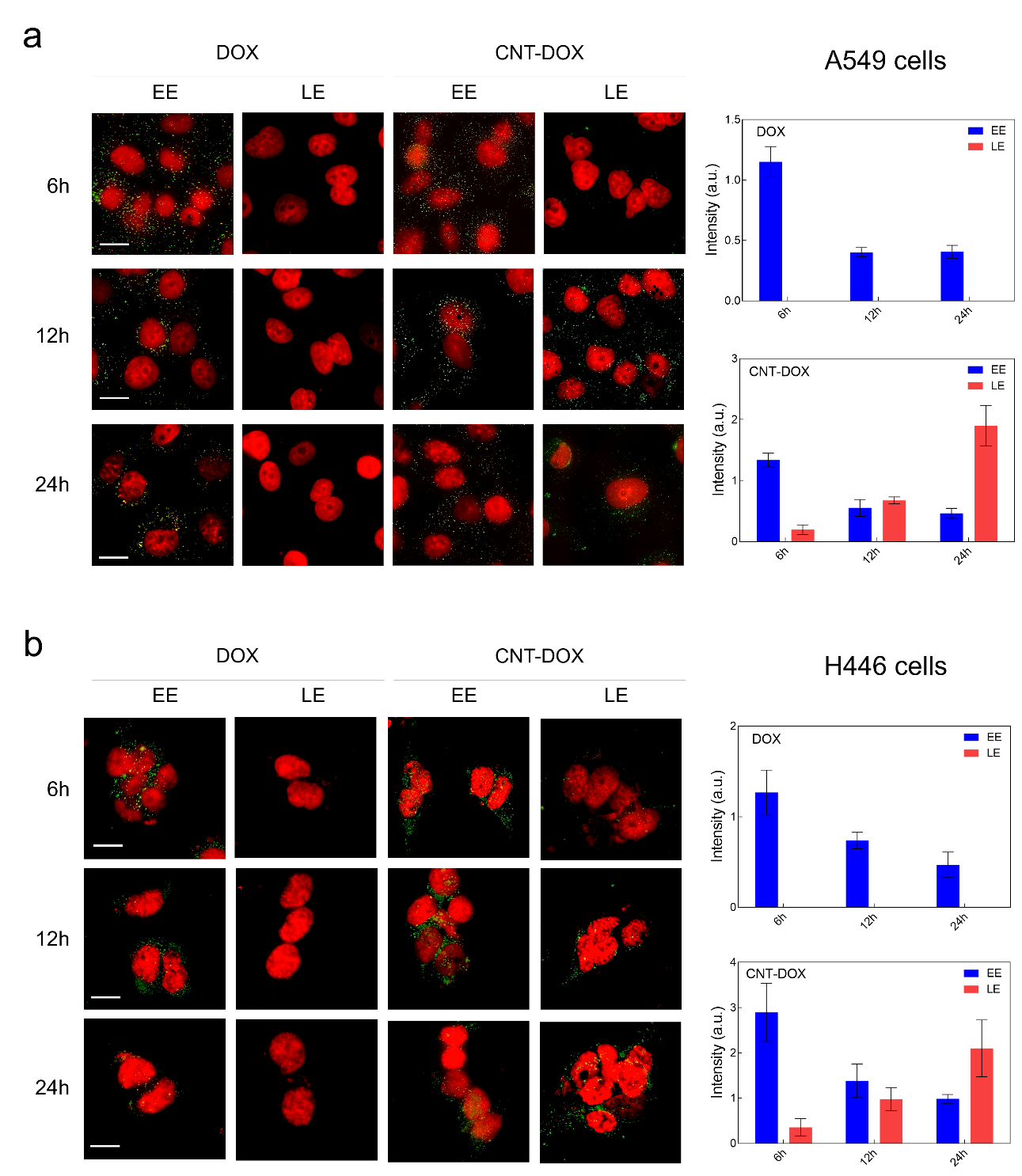


**Supplementary Fig. 5 Intracellular trafficking in normal lung cancer cells**

Confocal microscopy images and calibration bar graph visualizing DOX intensity (red) in the nuclei of (a) non-small cell lung cancer cells (A549 cells) and (b) small cell lung cancer cells (H446 cells), and the indicated vesicles (early endosomes (EEs) and late endosomes (LEs), green) after treatment with free DOX and CNT-DOX after 6, 12, and 24 h. Scale bar, 75 μm. Data represent the mean ± SEM (n = 6).

**
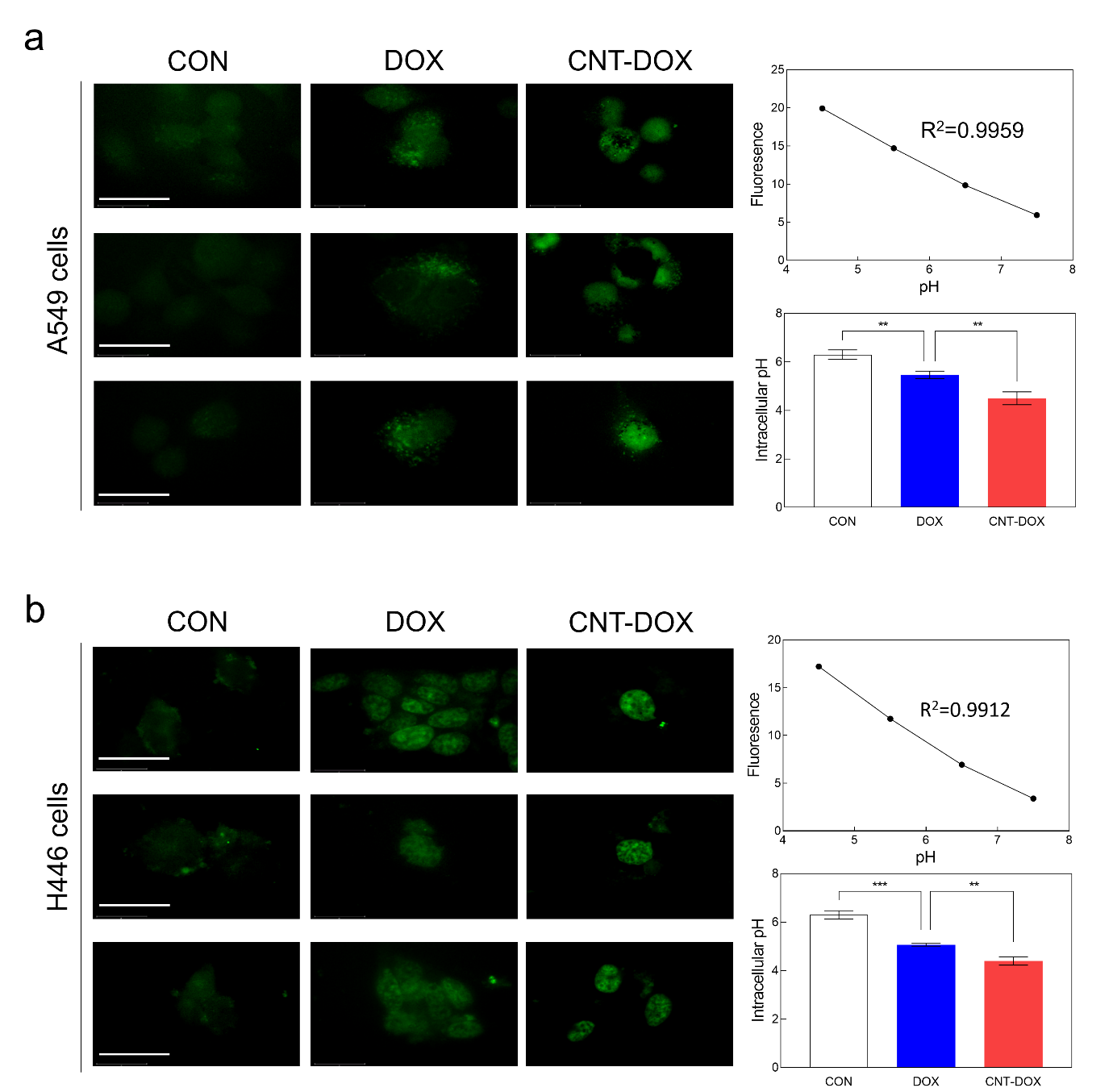
**

**Supplementary Fig. 6 Intracellular pH analysis in normal lung cancer cells**

Confocal images and calibration curve graph showing Rodo staining in (a) A549 cells and (b) H446 cells treated with DOX or CNT-DOX for 24 h. A549 and H446 cells treated with CNT-DOX showed more acidic conditions than those in the DOX-treated group. Scale bar, 75 μm. Data represent the mean ± SEM (n = 6). ***p* < 0.01 and ****p* < 0.001.


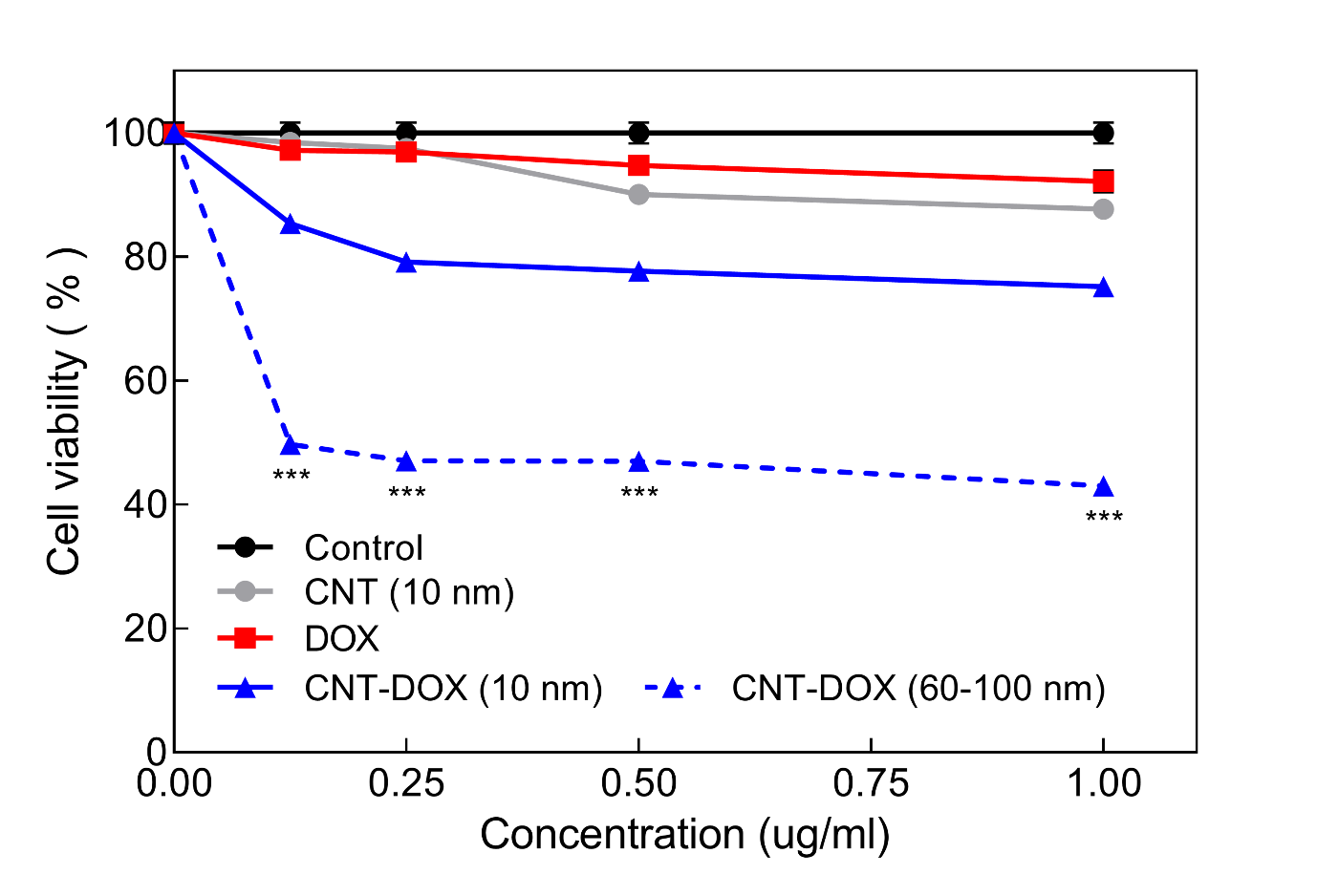


**Supplementary Fig. 7 Viability analysis**

Cell viability analysis using an MTT assay after treatment with CNT (10 nm), DOX, CNT-DOX (both 10 nm and 60-100 nm). CNT-DOX (60-100 nm) shows selective anticancer efficacy compared with other tested drugs (both CNT-DOX (10 nm) and DOX).

**
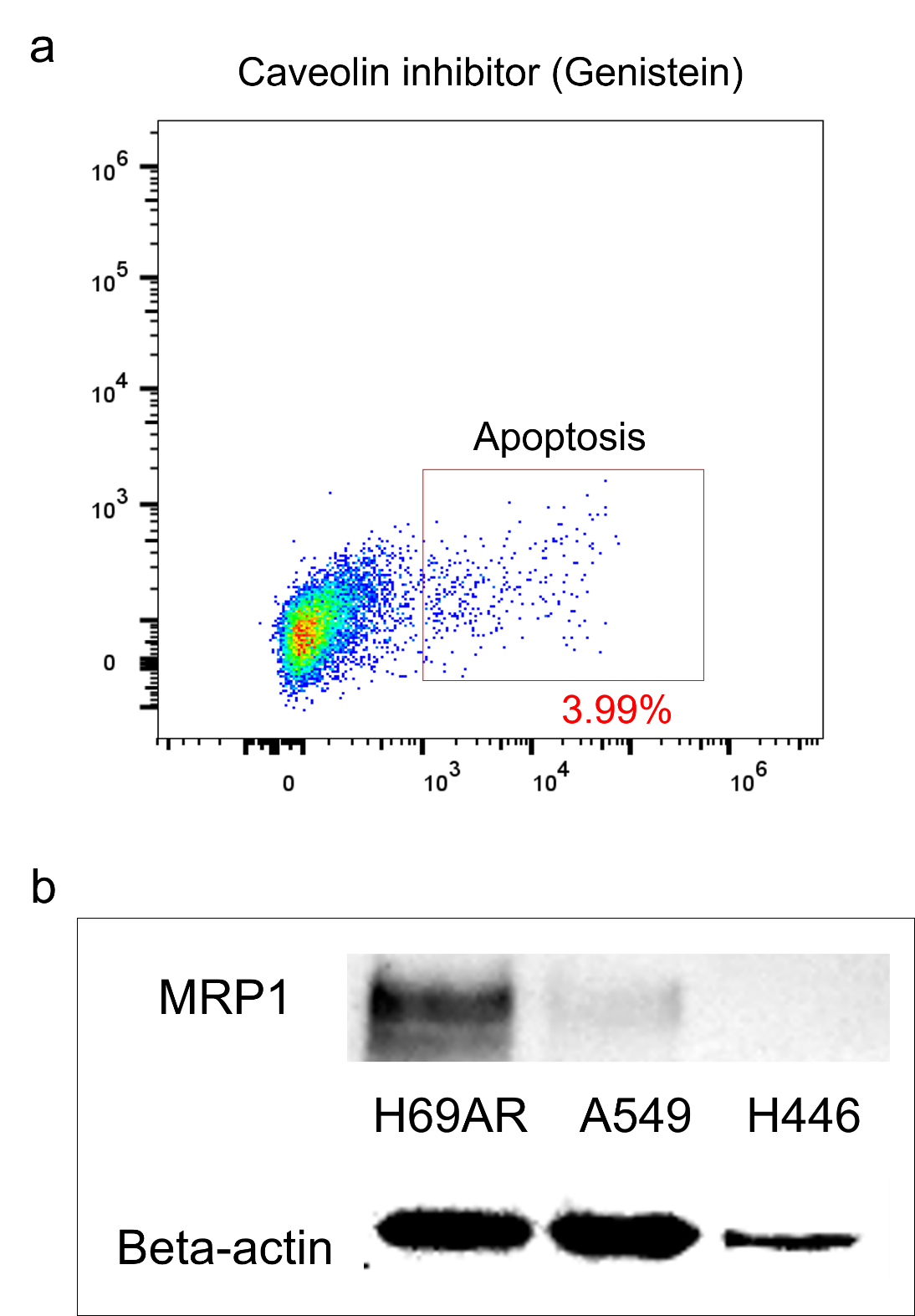
**

**Supplementary Fig. 8 Apoptosis and western blot analysis**

(a) Apoptosis analysis using a fluorescence-activated cell sorting (FACS) assay after treatment with GEN (caveolin endocytosis inhibitor) for 24 h. (c) Western blot analysis of mrp-1 expression in multidrug-resistant lung cancer cells (H69AR cells) and normal lung cancer cells (A549 and H446 cells).

**
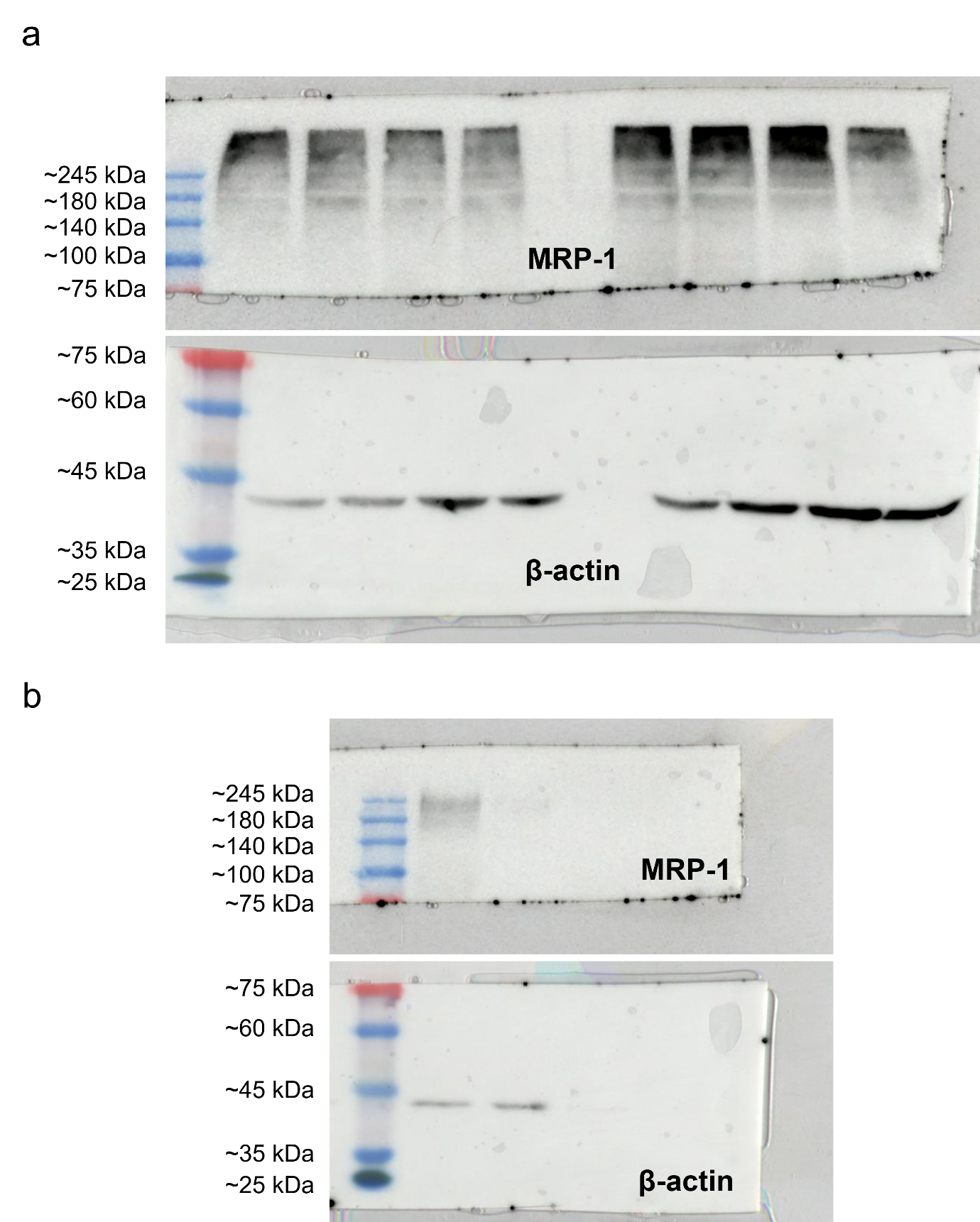
**

**Supplementary Fig. 9 The full-length staining of western blot analysis**

The full-length western blot analysis of (a) Fig. 7c and (b) Supplementary Fig. 3b.


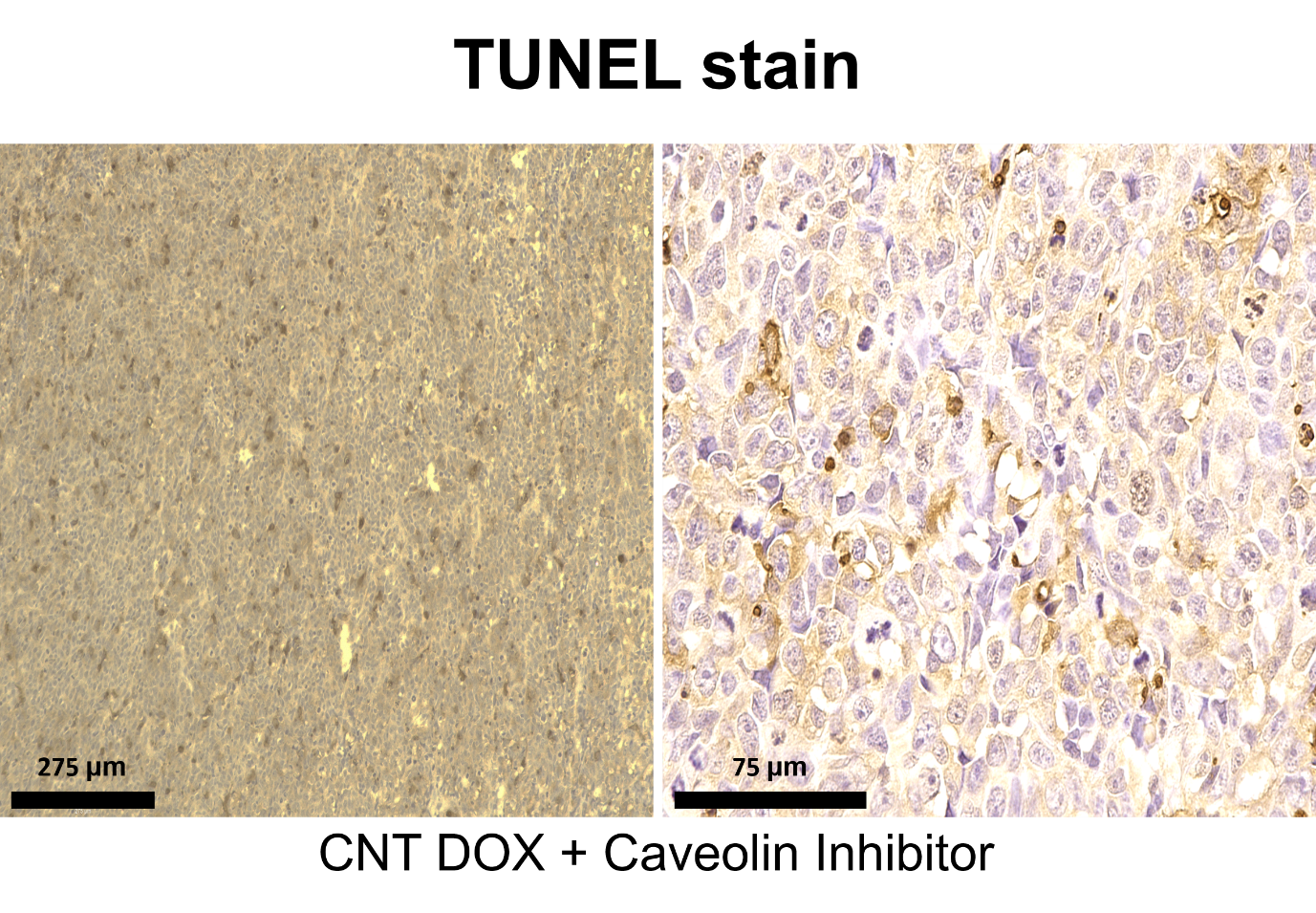


**Supplementary Fig. 10 Antitumor efficacy of xenograft mouse model**

Terminal deoxynucleotidyl transferase dUTP nick-end labeling (TUNEL) staining of H69AR tumor tissues after treatment with CNT-DOX and GEN (caveolin endocytosis inhibitor). Far-field (left) and near-field (right) images shows of apoptosis (brown colors in immunohistochemical staining).
